# Enhanced Oxygen Evolution Reaction Performance of NiMoO_4_/Carbon Paper Electrocatalysts in Anion Exchange Membrane Water Electrolysis by Atmospheric-Pressure Plasma Jet Treatment

**DOI:** 10.1021/acs.langmuir.4c03557

**Published:** 2024-11-01

**Authors:** Chen-Chen Chueh, Shuo-En Yu, Hsing-Chen Wu, Cheng-Che Hsu, I-Chih Ni, Chih-I Wu, I-Chun Cheng, Jian-Zhang Chen

**Affiliations:** † Graduate School of Advanced Technology, National Taiwan University, Taipei City 106319, Taiwan; ‡ Institute of Applied Mechanics, National Taiwan University, Taipei City 106319, Taiwan; § Department of Chemical Engineering, National Taiwan University, Taipei City 106319, Taiwan; ∥ Department of Electrical Engineering and Graduate Institute of Photonics and Optoelectronics, National Taiwan University, Taipei City 106319, Taiwan; ⊥ Advanced Research Center for Green Materials Science and Technology, National Taiwan University, Taipei City 106319, Taiwan

## Abstract

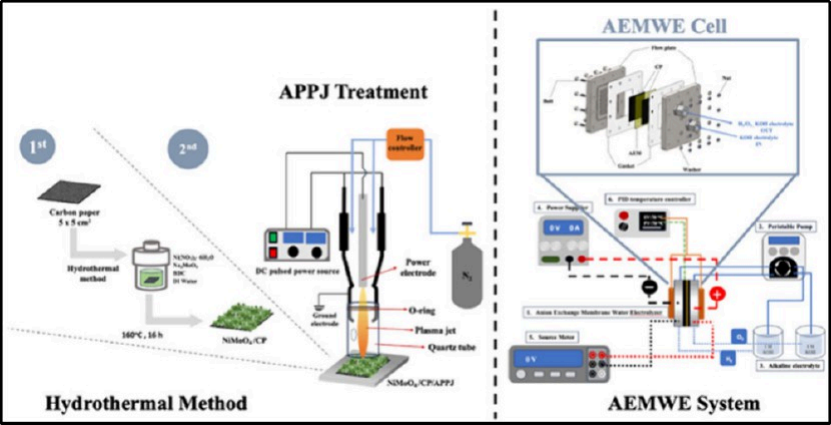

NiMoO_4_ was grown on carbon paper (CP) by a hydrothermal method. A rapid and high-temperature atmospheric-pressure plasma jet (APPJ) process was used to generate more oxygen-deficient NiMoO_4_ on the CP surface to serve as an electrode material for the oxygen evolution reaction (OER). After 60 s of APPJ treatment, the overpotential of the electrode at 100 mA/cm^2^ decreased to 790 mV and that at 10 mA/cm^2^ decreased to 368 mV. Additionally, the charge transfer resistance decreased from 2.8 to 1.2 Ω, indicating that APPJ treatment effectively reduced the electrode overpotential and impedance. The effect of NiMoO_4_/CP/APPJ-60 s on the anion exchange membrane water electrolysis (AEMWE) system was also tested. At a system temperature of 70 °C and current density of 100 mA/cm^2^, the energy efficiency reached 95.1%, and the specific energy consumption decreased from 4.02 to 3.83 kWh/m^3^. These results demonstrate that the APPJ-treated NiMoO_4_/CP electrode can effectively enhance the OER performance in water electrolysis and improve the energy efficiency of the AEMWE system. This approach shows promise in replacing precious metal electrodes, thereby potentially reducing the cost and providing an environmentally friendly alternative.

## 1 Introduction

As global warming becomes an increasingly serious problem, studies are actively investigating ways to reduce carbon emissions. Hydrogen energy is considered an emerging energy source that can replace fossil fuels.^1,2^ Hydrogen is categorized as gray, blue, or green hydrogen depending on its production method.^3,4^ Among these, green hydrogen has the lowest carbon emissions, making it one of the cleanest and most promising hydrogen energy technologies.^5^ Green hydrogen is produced by using biomass energy or renewable energy (such as wind, hydro, or solar) to generate electricity for performing water electrolysis. Water electrolysis primarily involves two reactions: the oxygen evolution reaction (OER) at the anode and the hydrogen evolution reaction (HER) at the cathode.

However, water electrolysis still has many challenges. Electrodes are typically made of precious metals such as ruthenium, platinum, and iridium as catalysts owing to their low overpotential.^6^ However, these precious metals are expensive and scarce, thus limiting the development of large-scale hydrogen production facilities. For hydrogen to become a mainstream energy source, these issues must be addressed. As noted above, water electrolysis involves two main reactions: HER and OER. In the half-reaction of the OER, four electrons need to be transferred to drive the reaction.^7^ Transition metal oxides have been found to reduce the reaction energy barrier and increase the electron transfer efficiency.^8,9^ In recent years, transition metal oxides such as NiFe_2_O_4_,^10^ NiCo_2_O_4_,^11,12^ CoMoO_4_,^13^ and NiMoO_4_^14^ have often been used as electrode materials for the OER.^15^ In particular, the nanowire structure of NiMoO_4_ provides a larger reactive surface area. In an alkaline environment, Ni ions rapidly convert to NiOOH that promote the OER reaction rate. Additionally, MoO_4_^2–^ can enhance the OER performance by improving the adsorption of OOH* intermediates.^16,17^

In recent years, plasma processes have been applied in various research fields and commercial applications. A key feature of plasma processing is its ability to rapidly enhance the hydrophilicity of materials,^18^ use ion bombardment to create pores and defects,^19^ or use plasma reactions and high temperatures to perform rapid annealing.^20^ Therefore, plasma processing is often considered an important process for improving the performance of energy devices such as supercapacitors and solar cells.^21,22^ Plasma processing has been used to perform ammonia doping for increasing the number of active sites on the electrode materials used for water electrolysis, thereby enhancing the HER performance.^23^ Alternatively, low-pressure plasma processing can be used to increase the number of oxygen vacancies in electrode materials, thereby improving the OER performance.^24,25^ The atmospheric-pressure plasma jet (APPJ) process differs from the traditional furnace process, which is time-consuming. The advantage of the APPJ post-treatment is its rapid thermal processing ability with the effect of reactive plasma species. Furthermore, no vacuum system is required with APPJ. Additionally, the APPJ process can be applied to large-scale manufacturing. The energy consumption of ultrafast APPJ treatment is estimated to be one-fifth that of a conventional furnace.^26^ Most recently, APPJ has been used for improving the performance of the electrodeposited NiFe electrocatalyst in anion exchange membrane water electrolysis (AEMWE).^27^

The three main types of water electrolysis approaches are alkaline water electrolysis (AWE), proton exchange membrane water electrolysis (PEMWE),^28^ and AEMWE.^29^ Among these, AWE is a mature technology that shows high stability and does not require precious metals as electrode materials. However, because no exchange membrane separates the cathode and anode reactions, the purity of the generated gases is not high and the operation current density is relatively low. Further, the bipolar pressure requires special control.^30^ In PEMWE, a proton exchange membrane separates the cathode and anode reactions. The electrolyte is typically an acidic solution, and precious metals are used as electrode materials in the cathode. This results in a high energy efficiency and higher-purity hydrogen gas. The disadvantage is the high cost and scarcity of the precious metals needed for use as the electrocatalyst. Furthermore, owing to the use of acidic electrolytes, using other metals might cause severe corrosion.^31^ AEMWE combines the advantages of AWE and PEMWE.^32^ It uses an alkaline electrolyte, nonprecious materials as electrocatalysts, and an anion exchange membrane to separate the cathode and anode reactions.^33^ This results in a higher current density and higher-purity hydrogen. Further, AEMWE is cost-effective and facilitates the development of large-scale hydrogen production systems.^34^

In this study, the transition metal oxide NiMoO_4_ was grown on carbon paper (CP) by using a hydrothermal method. A rapid APPJ treatment was then performed to modify the surface of the electrode material by creating surface pores and defects, thereby increasing the active surface area of the electrode to enhance the optical efficiency of the electrode to enhance the OER performance. The performance of the NiMoO_4_ electrocatalyst in an AEMWE system was evaluated.

## 2 Experimental Section

### 2.1 Chemicals and Materials

Sodium molybdate (Na_2_MoO_4_, 99%), nickel(II) nitrate (Ni (NO_3_)_2_·6H_2_O, 98%), potassium hydroxide (KOH, 85%), and deionized water were obtained from Thermo Scientific. All chemicals were used as obtained without any pretreatment. CP (thickness: 0.35 mm, CeTech, Taichung City, Taiwan) was cut into 5 × 5 cm^2^ pieces to serve as the porous transport layer substrate.

### 2.2 Electrocatalyst/Porous Transport Layer Preparation

#### 2.2.1 Hydrothermal Synthesis of NiMoO_4_

A NiMoO_4_/CP composite material was synthesized by using a hydrothermal method. CP (thickness: 0.35 mm) was used as the substrate, and NiMoO_4_ was grown in situ on it by a hydrothermal method. The hydrothermal solution was prepared by dissolving 25 mmol of Ni (NO_3_)_2_·6H_2_O, 25 mmol of Na_2_MoO_4_, and 7 mmol of terephthalic acid (BDC) in 160 mL of deionized water. After stirring the solution for approximately 1 h, the CP was immersed in a Teflon autoclave containing the solution. The autoclave was then placed in an oven and heated to 160 °C for 16 h. After the hydrothermal reaction was completed, the CP sample with NiMoO_4_ was removed, washed with deionized water, and sonicated in an ultrasonic bath. Finally, the sample was dried in an oven at 60 °C for 30 min to obtain the NiMoO_4_/CP composite material.^35,36^

#### 2.2.2 APPJ Surface Treatment of NiMoO_4_/CP

**Figure 1:**
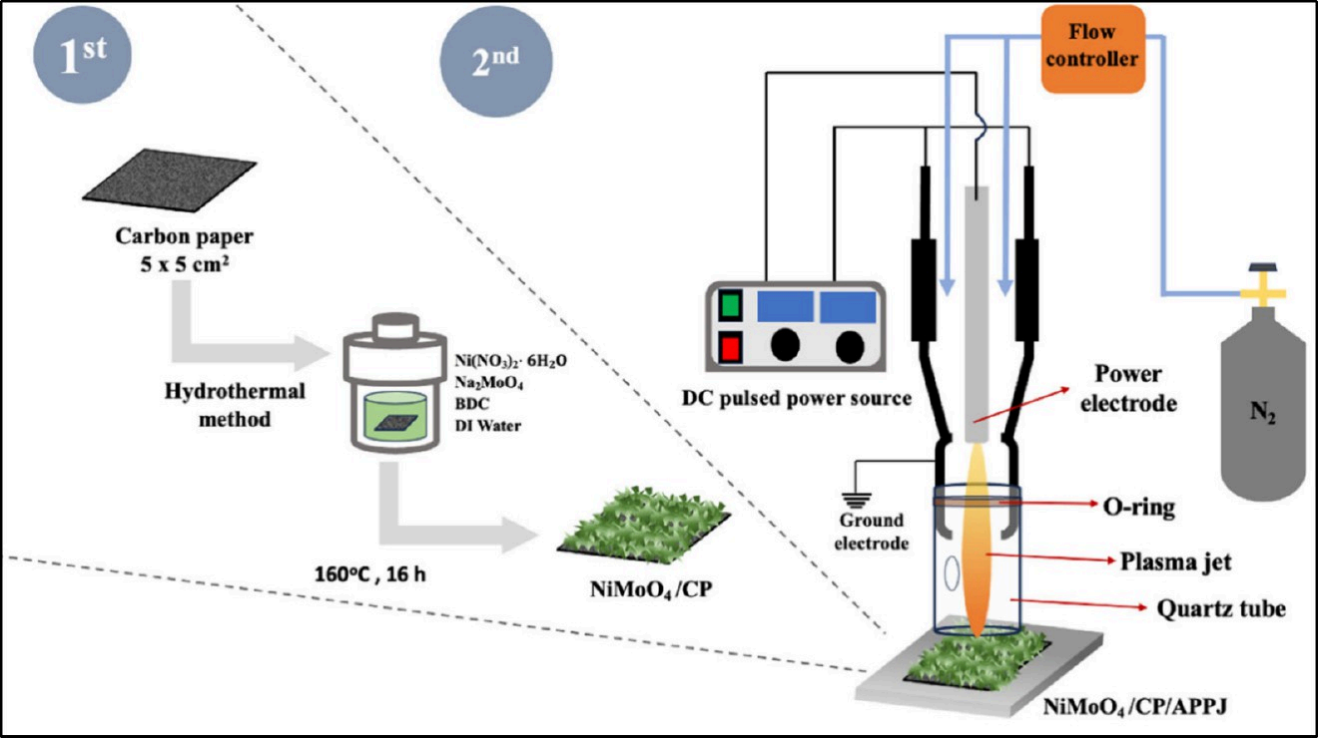
Hydrothermal method and APPJ process for producing NiMoO_4_/CP/APPJ.

The NiMoO_4_/CP was treated using a nitrogen APPJ under ambient pressure (1 atm) at a flow rate of 47 slm. The plasma temperature at this flow rate was approximately 500 °C. The distance between the electrode and APPJ quartz tube was 1 mm. As shown in Figure S1, the samples were treated with the APPJ for 30, 60, and 90 s.^23,37^ The resulting samples were respectively denoted as NiMoO_4_/CP-APPJ-30 s, NiMoO_4_/CP-APPJ-60 s, and NiMoO_4_/CP-APPJ-90 s; further, an untreated sample was denoted as NiMoO_4_/CP. Figure 1 shows the process flowchart for the electrode samples.

#### 2.2.3 Ru/CP/LPP-60 s Cathode Electrocatalyst Prepared by Hydrothermal Method and Low-Pressure Plasma Treatment

As in the growth of NiMoO_4_, CP was used as the cathode’s porous transport layer substrate. The solvothermal solution was prepared by using 5 mmol of RuCl_3_·3H_2_O, 80 mL of ethylene glycol, and 80 mL of deionized water. After stirring for 1 h, the CP was soaked in a Teflon autoclave containing the solution. The autoclave was then placed in the oven at 160 °C for 16 h. Subsequently, the Ru/CP was rinsed with deionized water, ultrasonicated, and heated in an oven at 60 °C for 30 min. Finally, it was processed by a low-pressure plasma for 60 s to improve its hydrophilicity. This sample, denoted as Ru/CP/LPP-60 s, was used as a cathode catalytic electrode in the AEMWE test.^25,38^

### 2.3 Materials Characterizations

Water contact angle analysis was performed using a goniometer (Sindatek, Model 100SB). The NiMoO_4_/CP was analyzed using scanning electron microscopy (SEM, JEOL JSM-7800F PRIME) and X-ray diffraction (XRD, Bruker D8 Discover). For XRD, we used a 2θ range of 5° – 75°. X-ray photoelectron spectroscopy (XPS, Thermo Scientific K-Alpha) was used to analyze the bonding states of the elements in the sample.

### 2.4 Electrochemical Measurements

All electrochemical measurements were performed using an electrochemical workstation (PGSTAT204, Metrohm, Utrecht, The Netherlands) at room temperature. The experimental setup consisted of a Ag/AgCl electrode (3 M KCl) as the reference electrode, a Pt electrode as the counter electrode, and a 1 × 3 cm CP-based working electrode prepared in this study. The electrolyte was 1 M KOH (pH = 14). Potential conversion was performed to ensure an accurate data analysis. All potentials were converted to the reversible hydrogen electrode (RHE) scale. According to the Nernst equation *E*_RHE_ = *E*_eap_ + *E*_ref_ + 0.059 × pH, where *E*_ref_ of the Ag/AgCl electrode was 0.197 V.^25,39^ We performed linear sweep voltammetry (LSV) measurements at a scan rate of 5 mV/s. The electrochemical double-layer capacitance (*C*_dl_) was determined using cyclic voltammetry (CV) at scan rates of 20, 50, 100, 150, 200, 250, and 300 mV/s in a potential window of 0.825 V–1.025 V (vs RHE). Electrochemical impedance spectroscopy (EIS) measurements were conducted in a fixed frequency range of 10 kHz to 0.1 Hz.

### 2.5 AEMWE

**Figure 2:**
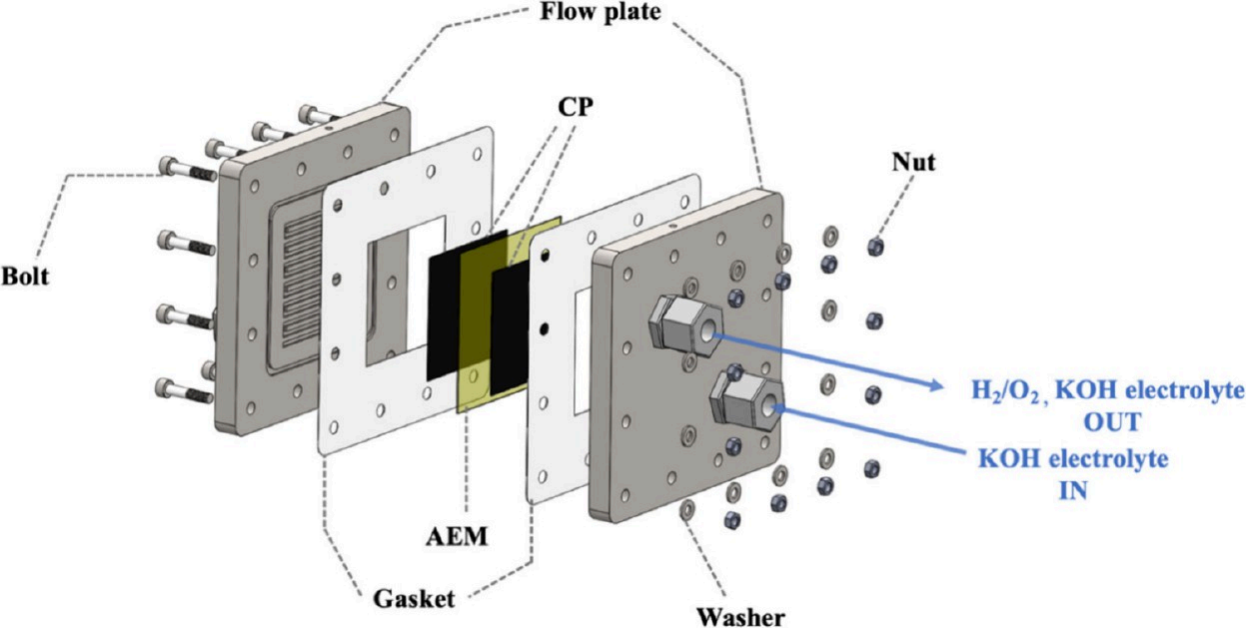
Schematic diagram of the AEMWE.

A commercial electrolysis module system (Figure 2) was purchased from Dioxide Materials. The outermost material was a nickel plate (11 cm × 11 cm) with flow channels. The electrocatalyst area was 5 cm × 5 cm. The electrocatalyst and porous transport layers (Ru/CP and NiMoO_4_/CP) were placed on the two flow plates separated by an anion exchange membrane (Fumasep FAA-3–50). The anion exchange membrane was soaked in 1 M KOH for 24 h. We used 1 M KOH as the electrolyte, and a peristaltic pump was used to pump the KOH electrolyte into the nickel flow plate at a flow rate of 10 mL/min. A power supply (SPS-1230, GWInstek, New Taipei City, Taiwan) was used to supply the voltage and current, and a multimeter (15B, FLUKE, Everett, WA, USA) was used to measure the actual voltage of the module. For temperature control, we used a PID temperature controller (DTA4848 V1, Delta Electronics, Inc., Taipei City, Taiwan) to connect a heating plate (100 W, 110 V, approximately 10 cm × 10 cm) to the nickel plate. A K-type thermocouple was used to measure the temperature of the nickel flow plate. The system is shown in Figure S2. For safety and module protection, the maximum power supply voltage was controlled below 2 V.

## 3 Results and Discussion

**Figure 3:**
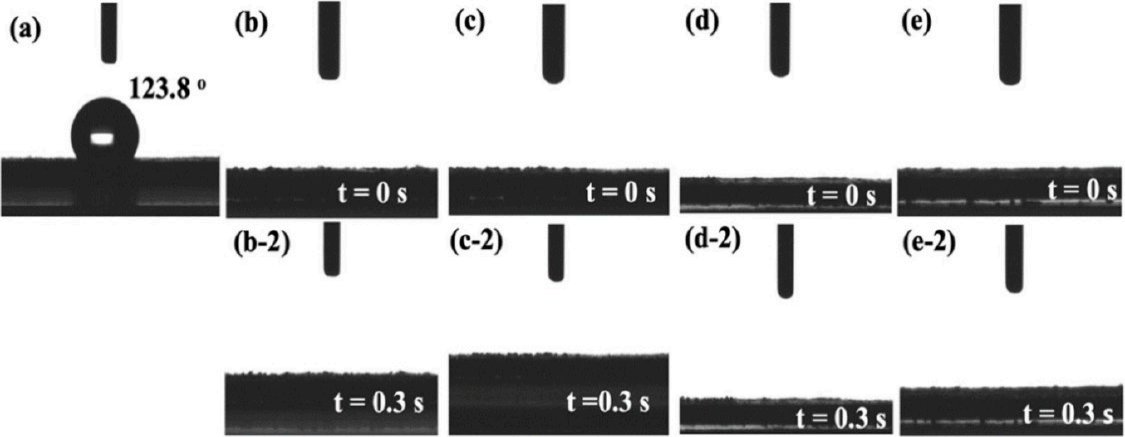
Water contact angle test: (a) CP, (b-1,2) NiMoO_4_/CP, (c-1,2) NiMoO_4_/CP/APPJ-30 s, (d-1,2) NiMoO_4_/CP/APPJ-60 s, and (e-1,2) NiMoO4/CP/APPJ-90 s.

**Figure 4:**
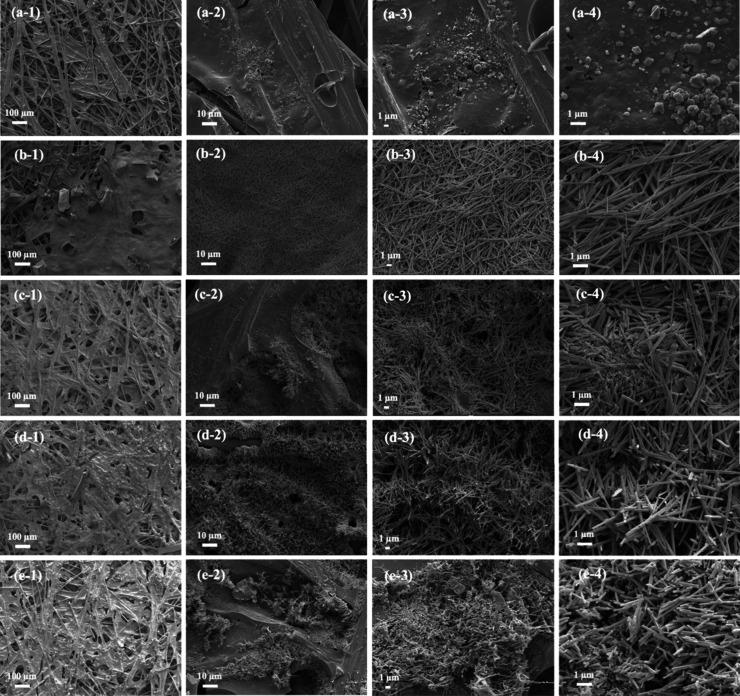
SEM images with 100×, 1000×, 3000×, and 10 000× magnification: (a) CP, (b) NiMoO_4_/CP, (c) NiMoO_4_/CP/APPJ-30 s, (d) NiMoO_4_/CP/APPJ-60 s, and (e) NiMoO_4_/CP/APPJ-90 s.

Water contact angle measurement is a common method to assess the surface hydrophilicity of materials. Hydrophilicity is a crucial property for electrocatalytic materials, as it influences the interaction between the electrolyte and the catalyst surface. Enhanced hydrophilicity promotes intimate contact between the electrolyte and the catalyst, thereby facilitating bubble detachment and increasing the electrolyte-electrocatalyst reaction area and in turn improving the catalytic performance.^40^ APPJ has been shown to be a good approach to improve hydrophilicity.^41^Figure 3 shows the water contact angles of CP, NiMoO_4_/CP, NiMoO_4_/CP/APPJ-30 s, NiMoO_4_/CP/APPJ-60 s, and NiMoO_4_/CP/APPJ-90 s. CP appeared hydrophobic, with a water contact angle of 123.8°; in all other samples, water droplets immediately penetrated the electrocatalyst/porous transport layer. After APPJ treatment, the electrocatalytic electrode remained hydrophilic; Therefore, the electrode and electrolyte still maintain good contact, and the bubble detachment rate remains satisfactory. As a result, the electrodes can efficiently electrolyze water to produce hydrogen or oxygen.^24^ The surface morphology of CP, NiMoO_4_/CP, NiMoO_4_/CP/APPJ-30 s, NiMoO_4_/CP/APPJ-60 s, and NiMoO_4_/CP/APPJ-90 s was observed using SEM Figure 4(a) shows that the CP fibers themselves have pores. Figure 4(b) shows that after in situ hydrothermal growth, NiMoO_4_ nanorods with a length of approximately 3–5 μm are seen to be deposited on the CP.^42^Figures 4(c)–(e) show the NiMoO_4_/CP/APPJ-30 s, NiMoO_4_/CP/APPJ-60 s, and NiMoO_4_/CP/APPJ-90 s samples treated with APPJ for different durations. Defects produced by ion bombardment can be observed on the surface. These plasma-induced defects can create a larger active surface area, thereby enhancing the catalytic effect.^25,43^Figure S3 shows the SEM-EDS elemental mapping images of NiMoO_4_/CP/APPJ-60 s at 3000× magnification.

**Figure 5:**
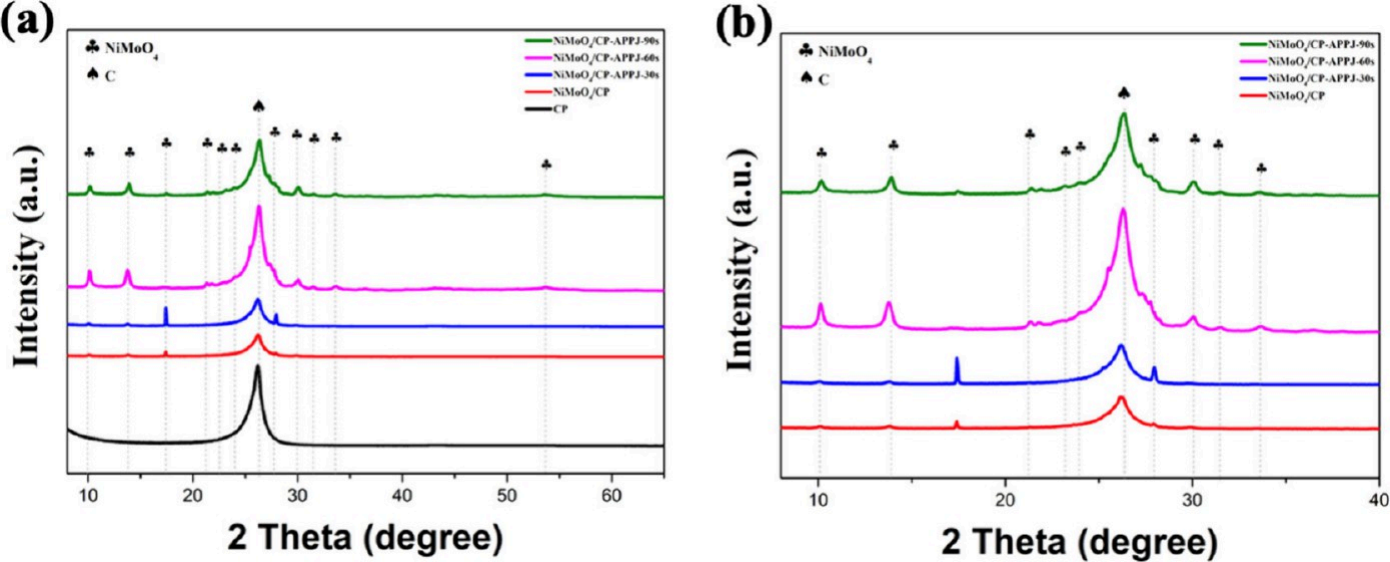
XRD patterns: (a) carbon paper, NiMoO_4_/CP, NiMoO_4_/CP/APPJ-30 s, NiMoO_4_/CP/APPJ-60 s, and NiMoO_4_/CP/APPJ-90 s. (b) NiMoO_4_/CP, NiMoO_4_/CP/APPJ-30 s, NiMoO_4_/CP/APPJ-60 s, and NiMoO_4_/CP/APPJ-90 s zoom-in XRD patterns.

**Figure 6:**
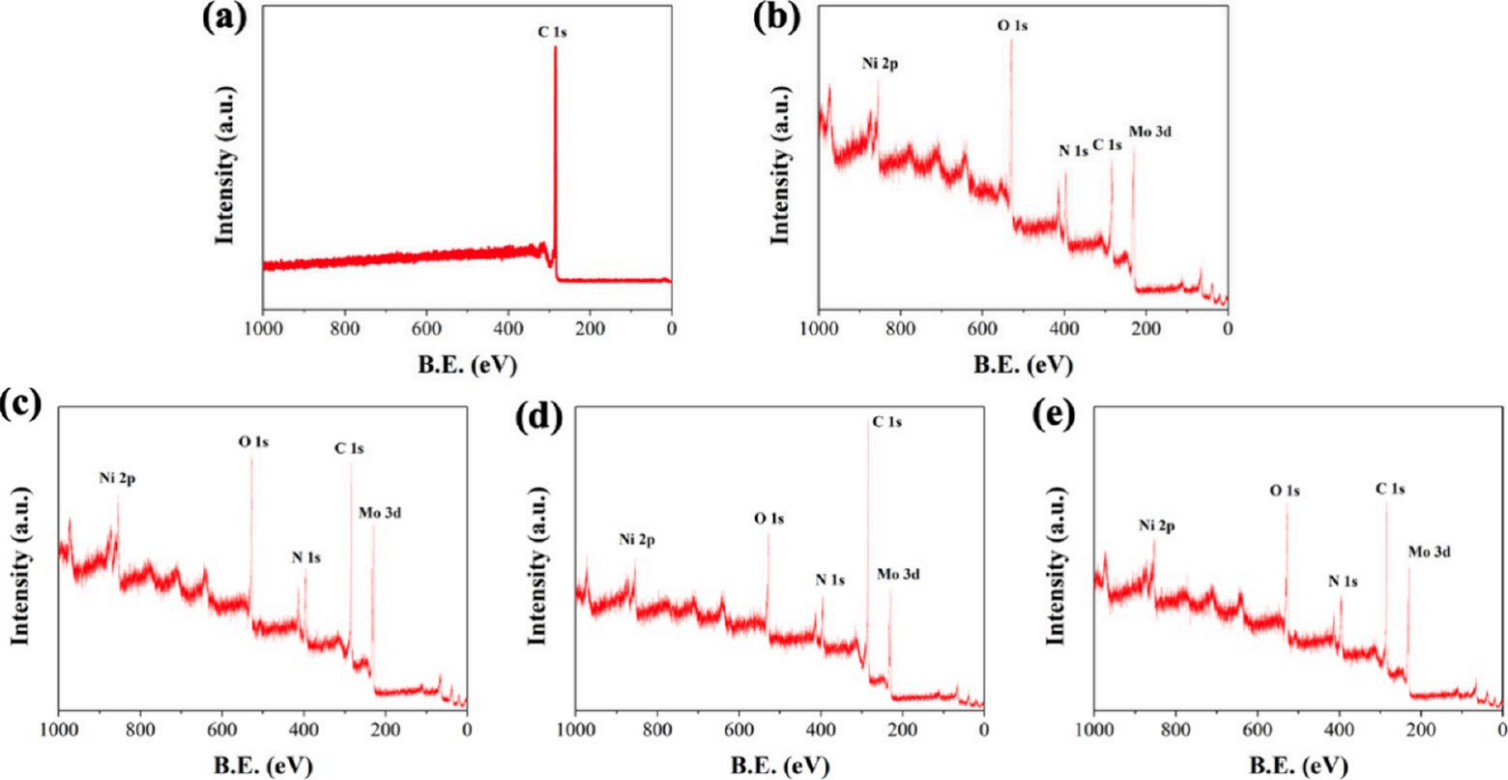
Survey XPS spectra: (a) CP, (b) NiMoO_4_/CP, (c) NiMoO_4_/CP/APPJ-30 s, (d) NiMoO_4_/CP/APPJ-60 s, and (e) NiMoO_4_/CP/APPJ-90 s.

**Figure 7:**
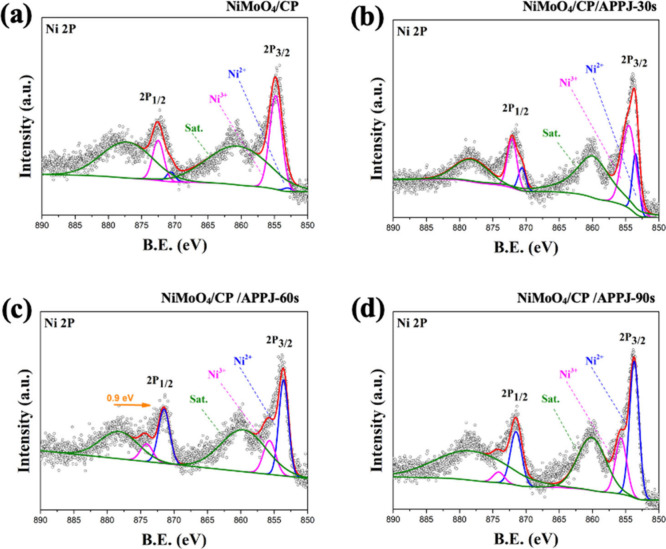
HRXPS spectra of Ni 2p: (a) NiMoO_4_/CP, (b) NiMoO_4_/CP/APPJ-30 s, (c) NiMoO_4_/CP/APPJ-60 s, and (d) NiMoO_4_/CP/APPJ-90 s.

**Figure 8:**
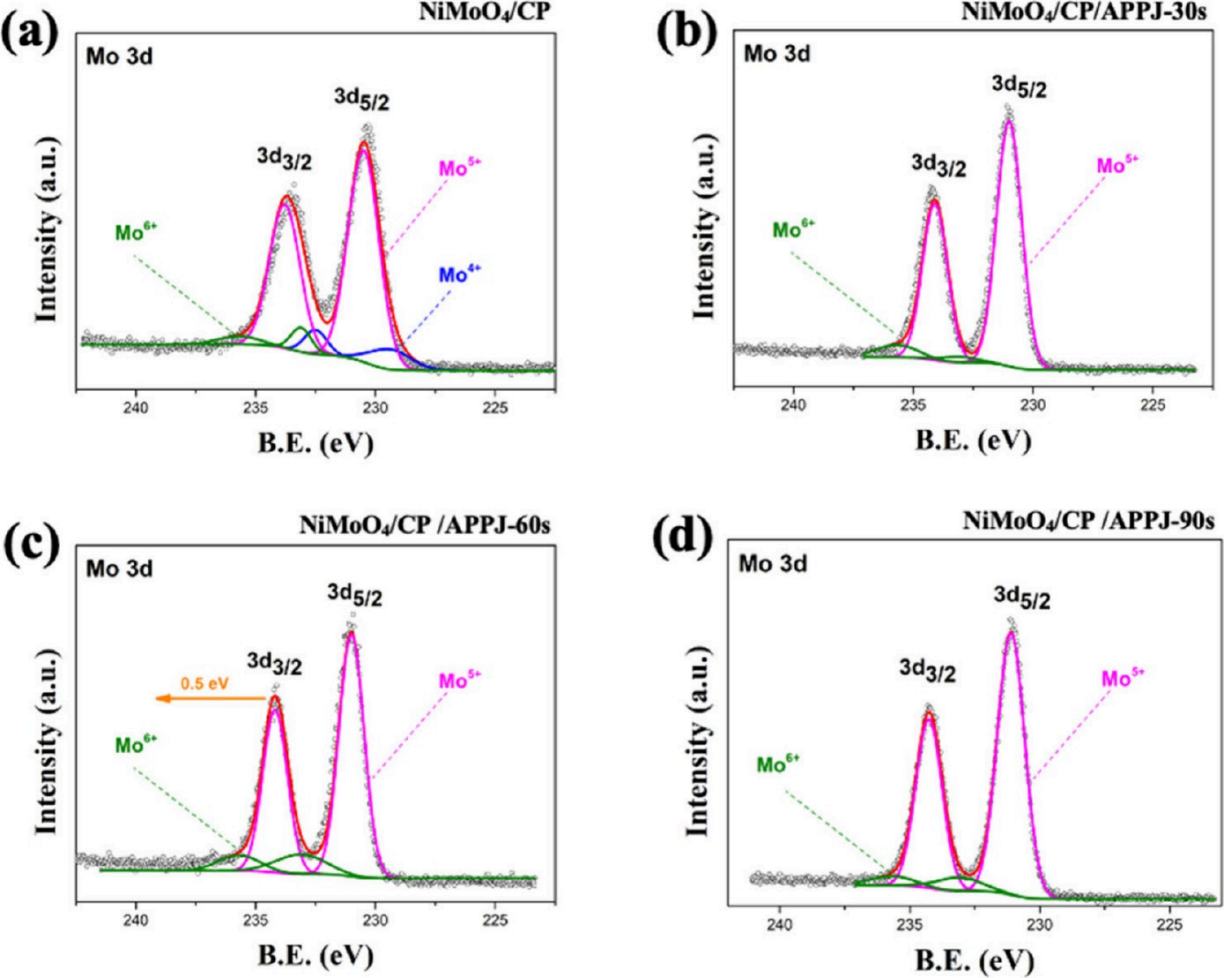
HRXPS spectra of Mo 3d: (a) NiMoO_4_/CP, (b) NiMoO_4_/CP/APPJ-30 s, (c) NiMoO_4_/CP/APPJ-60 s, and (d) NiMoO_4_/CP/APPJ-90 s.

Figure 5(a) shows the XRD patterns of CP, NiMoO_4_/CP, NiMoO_4_/CP/APPJ-30 s, NiMoO_4_/CP/APPJ-60 s, and NiMoO_4_/CP/APPJ-90 s. The crystal plane signals of NiMoO_4_/CP prepared by the hydrothermal method are not yet prominent. After APPJ treatment, stronger NiMoO_4_ peaks can be observed at 2θ values of 10°, 14°, 21°, 23°, 24°, 27°, 30°, 31°, 34°, and 53.4°.^44,45^ This indicates that NiMoO_4_ was successfully grown on the CP surface by using the hydrothermal method.^17,46,47^ The partially enlarged XRD patterns are shown in Figure 5(b). Moreover, under the effects of the high temperature and plasma in the rapid APPJ process, NiMoO_4_ crystals form more completely on the CP. Among the various samples, the sample treated with APPJ for 60 s shows the strongest signal. XPS measurement is an effective method for analyzing the bonding types and chemical configurations of materials in samples. Figures 6(a)–(e) show the XPS survey spectra of CP, NiMoO_4_/CP, NiMoO_4_/CP/APPJ-30 s, NiMoO_4_/CP/APPJ-60 s, and NiMoO_4_/CP/APPJ-90 s. Figures 6(c)–(d) indicate that the signals of Ni, Mo, and O are still present after APPJ treatment. Figures 7(a)–(b) show high-resolution XPS (HRXPS) of Ni 2p. The binding energies of 853.3 and 871.2 eV respectively correspond to Ni (II) 2p_3/2_ and Ni (II) 2p_1/2_, and those of 855.7 and 874.1 eV respectively correspond to Ni (III) 2p_3/2_ and Ni (III) 2p_1/2_. Additionally, the peaks at 859.6 and 878.2 eV are satellite peaks of Ni 2p_3/2_ and Ni 2p_1/2_ respectively.^48−50^Table S2 shows the area proportions of various configurations of Ni 2p. The results indicate that the proportion of the Ni^2+^ area increases with a longer APPJ treatment time. For NiMoO_4_/CP/APPJ-60s, the Ni^2+^ area accounts for 30.04%. Figure 8 shows the HRXPS of Mo 3d. The binding energies of 229.4 and 232.5 eV respectively correspond to Mo (IV) 3d_5/2_ and Mo (IV) 3d_3/2_, and those of 230.5, 234.1, 233.1, and 235.7 eV respectively correspond to Mo (V) 3d_5/2_, Mo (V) 3d_3/2_, Mo (VI) 3d_5/2_, and Mo (VI) 3d_3/2_.^11,51^Table S3 shows the area proportions of different valence states of Mo 3d in HRXPS. Mo^6+^ shows a maximum area proportion of 13.65% when the APPJ treatment time is 60 s. The HRXPS results of Ni 2p and Mo 3d together indicate that Ni and Mo form different types of oxides attached to the CP surface through in situ hydrothermal growth. After APPJ treatment, under the rapid processing and ion bombardment, the oxidation states of Ni^2+^ and Mo^6+^ increased, resulting in the synthesis of more NiMoO_4_ on the CP.^52,53^ In addition, the samples treated with APPJ for 60 s showed the largest binding energy shifts in Ni 2p and Mo 3d, at negative 0.9 eV and positive 0.5 eV, respectively. This indicates that the 60 s of APPJ treatment resulted in the most significant changes in the metal valence states.

**Figure 9:**
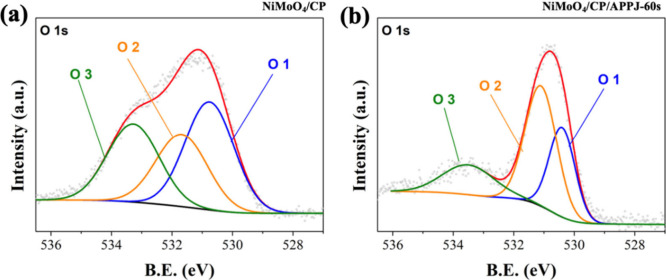
HRXPS spectra of O 1s: (a) NiMoO_4_/CP and (b) NiMoO_4_/CP/APPJ-60 s.

Figure 9 shows the HRXPS image of the O 1s. The binding energy of 530.4 eV corresponds to the M-O-M peak, indicating the lattice oxygen signal (labeled as O 1). Additionally, the peak at 531.3 eV corresponds to oxygen vacancies (labeled as O 2), and that at 533.5 eV corresponds to surface-adsorbed water (labeled as O 3).^50,54^Figure 9(b) shows that NiMoO_4_/CP treated with APPJ for 60 s has a higher proportion of oxygen vacancies. Table S3 shows the area proportions of the 1s HRXPS results. The proportion of oxygen vacancies increases by approximately 20% after APPJ treatment. The oxygen vacancies may be generated owing to ion bombardment from the plasma, and the creation of these oxygen vacancies helps to enhance the OER reaction, thus increasing the efficiency of water electrolysis.^13,37,55,56^Figure S4 and Table S5 show the HRXPS of C 1s and the area proportions of various bonds in HRXPS, respectively. The C=C peak is at 284.5 eV; metal carbide peak is at 282.5 eV; and C–C, C–O, and carbonyl group peaks are at 285.5, 286.3, and 288.1 eV, respectively.^49,57^ The peaks of metal carbides can be observed in NiMoO_4_/CP grown in situ by the hydrothermal method. We speculate that BDC is formed by a combination of the oxidation states of Ni and Mo. After the high-temperature and rapid APPJ treatment, it is converted into amorphous carbon. Amorphous carbon can form a protective layer to enhance the stability of the electrode.^39^ Overall, the XRD and XPS results indicate that different types of Ni and Mo oxides are grown in situ by the hydrothermal method. After the high-temperature and rapid APPJ processing and ion bombardment, more NiMoO_4_ is generated on the CP to improve the OER effect, and converting the organic ligand into a carbon protective layer increases the mechanical strength of the electrode and creates more oxygen defects to improve the efficiency of water electrolysis.

**Figure 10:**
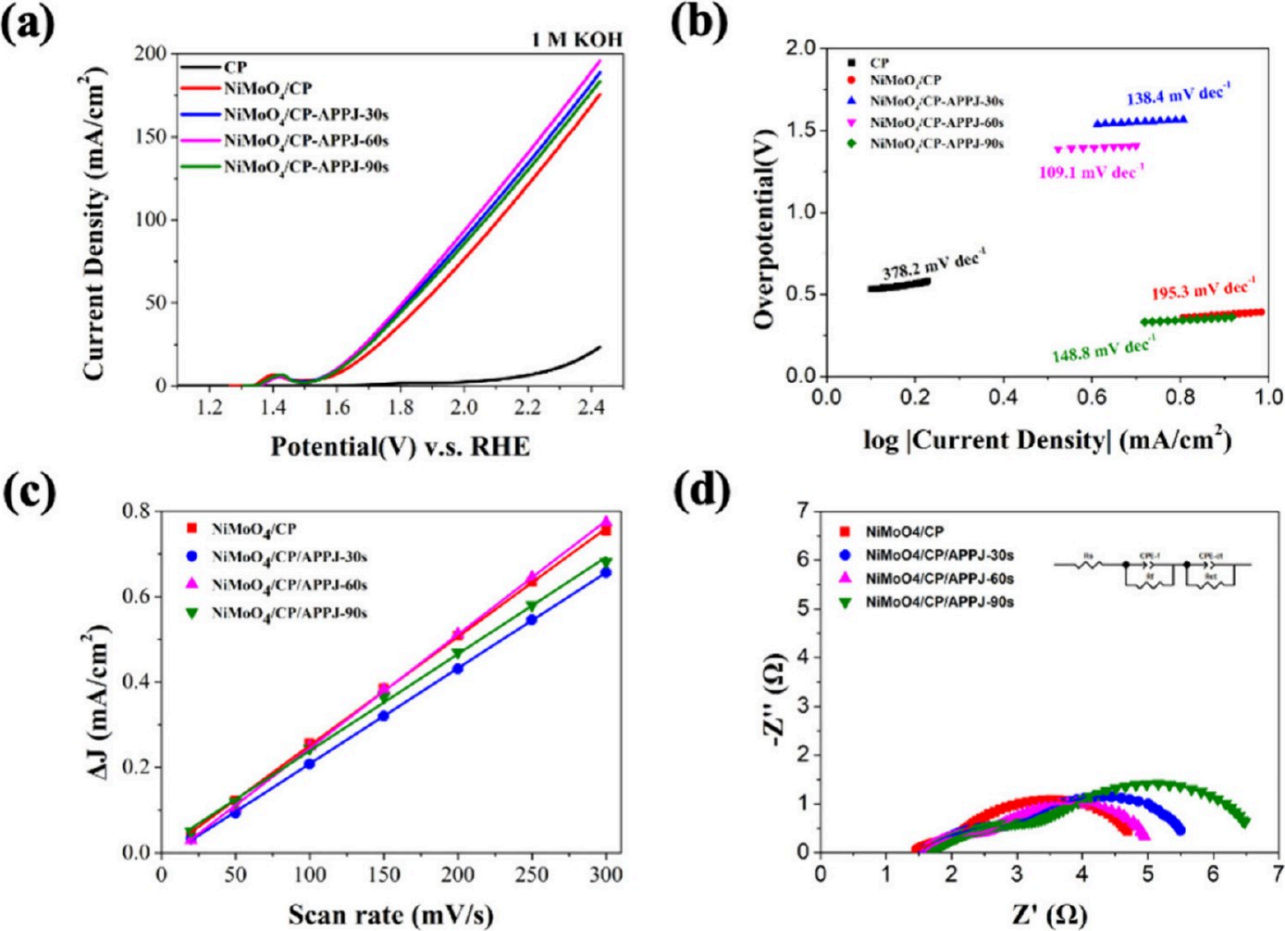
Electrochemical characterization measurement results: (a) LSV polarization curves and (b) Tafel slope plots. (c) EDLC of different electrodes. (d) Nyquist plots at an overpotential of 350 mV versus RHE.

Electrochemical measurements and analysis were performed using LSV at a scan rate of 5 mV/s. The electrolyte used was 1 M KOH, and measurements were conducted by using a three-electrode method. The electrode performing OER needs to transfer four electrons and overcome the energy barrier.^58,59^Figure 10(a) and Table 1 show the LSV polarization curves and the overpotential at 100 mA/cm^2^, respectively. The results indicate that APPJ-treated NiMoO_4_/CP samples all exhibit lower overpotentials, with the 60 s APPJ treatment resulting in the lowest value of 790 mV. Additionally, a distinct oxidation peak appears at 1.4 V (vs RHE) owing to the conversion of surface Ni^2+^ to Ni^3+^ resulting in the formation of nickel (oxy)hydroxide (NOOH).^35,60^Figure 10(b) shows the Tafel slope values under APPJ processing at different speeds per second. The lower the Tafel slope, the lower is the voltage required to generate current.^61^ NiMoO_4_/CP/APPJ-60s shows the lowest Tafel slope value of 109.1 mV/dec The LSV and Tafel slope results are consistent with the material analysis ones. The transition metal oxide NiMoO_4_ improves the OER effect.^62^ The generation of more NiMoO_4_ and oxygen defects under the APPJ treatment also contributes to the OER effect.

**Table 1:** Electrochemical Measurement Parameters for Each Electrocatalyst

**Electrocat**a**lyst**	**Overpotential (mV) @100 mA/cm**^**2**^	**Tafel slope (mV/dec)**	***R***_**ct**_**(Ω)**	**2*C***_**dl**_**(mF/cm**^**2**^**)**
CP	-	378.2	29.7	-
NiMoO_4_/CP	879	195.3	2.8	2.54
NiMoO_4_/CP/APPJ-30 s	810	138.4	1.9	2.24
NiMoO_4_/CP/APPJ-60 s	790	109.1	1.2	2.67
NiMoO_4_/CP/APPJ-90 s	836	148.8	2.4	2.27

The electrochemical surface area can be used to evaluate the surface characteristics of the electrode. The electric double layer capacitance (EDLC) is proportional to the electrochemical active surface area.^63^Figure 10(c) shows that NiMoO_4_/CP, NiMoO_4_/CP/APPJ-30s, NiMoO_4_/CP/APPJ-60s, and NiMoO_4_/CP/APPJ-90s were measured in the non-Faradaic zone at different scanning rates.^64^ The calculated EDLC in Table 1, indicates that, compared to the NiMoO_4_ grown by the hydrothermal method, the NiMoO_4_ treated with APPJ for 60 s has the largest EDLC. Therefore, the APPJ-treated sample has a larger electrochemically active surface area. Additionally, for the EIS measurement, we measured the Nyquist plot data under an overpotential of 350 mV and used an appropriate equivalent circuit to fit the charge transfer impedance. In Figure 10(d), two semicircles can be observed. The first semicircle is related to the porous structure of the electrode itself, representing the *R*_f_ value produced when the electrolyte fills the pores. The second semicircle represents the charge transfer impedance between the electrode and the electrolyte.^65−67^ Among these, the NiMoO_4_/CP treated with APPJ for 60 s has the lowest charge transfer impedance of 1.9 Ω, which is significantly lower than the charge transfer impedance of 2.8 Ω for the in situ grown NiMoO_4_/CP. The results of the EDLC and EIS measurements show that after APPJ treatment, NiMoO_4_/CP has a larger electrochemically active surface area owing to plasma ion bombardment, while simultaneously reducing the charge transfer impedance between the electrolyte and the electrode.^25,37^ Stability is one of the important indicators for the OER. It is crucial for the electrode to maintain the same catalytic effect over a long period. Figure S5 shows the chronopotential test of NiMoO_4_/CP/APPJ-60 s for 24 h at 10 mA/cm^2^. The results demonstrate its stability and show that under constant current, the electrode maintains a stable overpotential of approximately 330 mV. Figure S6 shows a comparison of the LSV polarization curves of NiMoO_4_/CP/APPJ-60 s before and after the 24 h stability measurement. At a current density of 100 mA/cm^2^, the overpotential increases by approximately 2%. Additionally, some articles mention that the nanosheet structure of NiMoO_4_, grown in situ via hydrothermal methods exhibits increasingly better catalytic properties after undergoing cyclic voltammetry and chronoamperometry tests.^68^ Other studies suggest that when NiMoO_4_ is used as a catalytic electrode, FT-IR and XPS analyses reveal that, during the OER process, molybdenum facilitates the formation of more nickel into NiOOH, which then disappears from the surface. This structural change supports prolonged reactions during the overall OER process.^69^ Long-term stability and durability of NiMoO_4_ are critical issues for practical electrocatalyst application. This will require further in-depth investigation for APPJ-processed NiMoO_4_ in the future. The OER electrocatalyst performance in alkaline solution is compared with those in literatures, as shown in Table S1.

**Figure 11:**
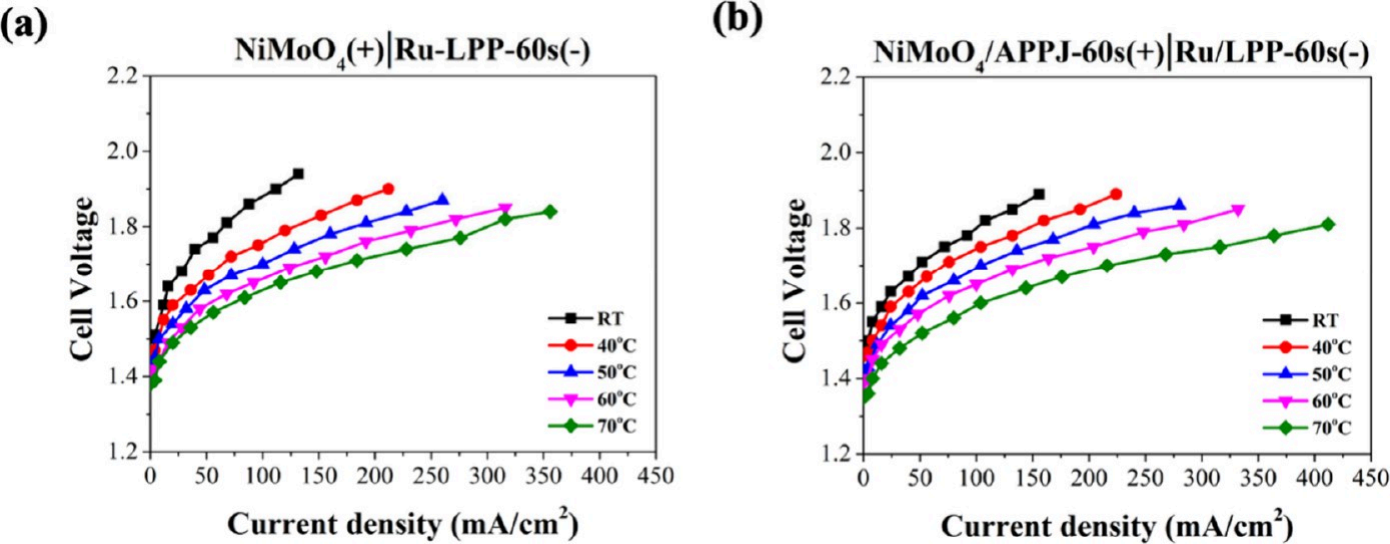
Cell voltage and current density curves in the AEMWE at different temperature: (a) NiMoO_4_/CP(+)|Ru/CP/LPP-60 s(−) and (b) NiMoO_4_/CP/APPJ-60 s (+)|Ru/CP/LPP-60 s(−).

**Figure 12:**
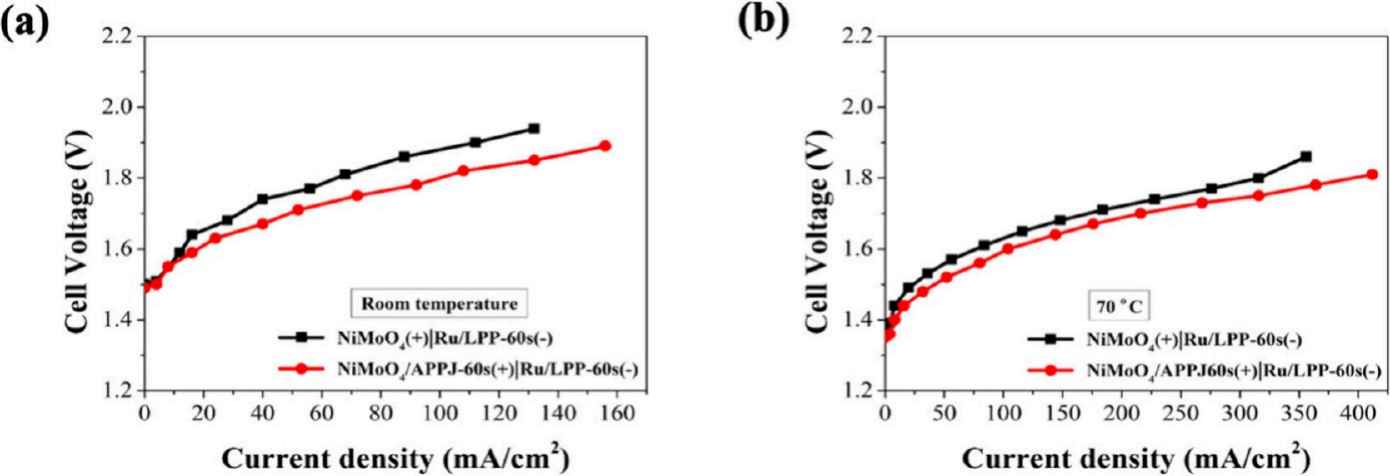
Comparison of different anode electrocatalysts in AEMWE: (a) room temperature and (b) 70 °C.

The electrode made from NiMoO_4_/CP by rapid APPJ processing contributes to improved OER performance; therefore, it was applied in an AEMWE system for a more practical application. In the AEMWE system, we used NiMoO_4_/CP and NiMoO_4_/CP/APPJ-60 s as the anodes for the OER and Ru/CP/LPP-60 s as the cathode for the HER. Measurements were taken at power supply voltages below 2 V, and voltage–current curves were recorded at room temperature, 40 °C, 50 °C, 60 °C, and 70 °C. The hydrogen production rate at fixed current densities was measured using the water displacement method, and the efficiency was calculated. Figure 11 shows the cell voltage–current curves at different temperatures, and Figure S7 shows the applied voltage–current curves at different temperatures. As the temperature increases, the current at the same voltage increases. Additionally, Figure 12 and Figure S8 show that after 60 s of APPJ treatment, NiMoO_4_/CP exhibits increased current at the same voltage. Table 2 shows the hydrogen production rates and calculated efficiency of the electrodes at different current densities and temperatures. The energy efficiency^38^ is used to evaluate how much of the input electrical power can be converted into hydrogen energy, expressed as thermal energy. *I* is the current applied to the electrodes, *V*_ps_ is the voltage input from the power supply, and *P*_H_2__ is the volume of hydrogen gas produced per minute in milliliters, and each milliliter of hydrogen gas can produce 11.7 J of energy.

**Table 2:** Efficiency of AEMWE at Different Current Densities and Temperatures

Electrocatalysts	Temperature	*V*_apply_	*V*_cell_	Current density	H_2_ production rate (experimental)	Specific energy consumption	Specific energy consumption	Energy efficiency η
Unit	°C	V	V	mA/cm^2^	mL/min	KWh/m^3^	KWh/kg	%
NiMoO_4_/CP(+)| Ru/CP/LPP-60 s(−)	RT	1.93	1.88	100	20	4.02	45	80.8
	70	1.68	1.63	100	20	3.5	39.2	92.8
		1.82	1.72	200	37	4.09	45.9	79.3
		1.93	1.79	300	57	4.23	47.4	76.8
NiMoO_4_/CP/APPJ-60s(+)| Ru/CP/LPP-60 s(−)	RT	1.84	1.8	100	20	3.83	42.93	84.8
	70	1.64	1.6	100	20	3.41	38.26	95.1
		1.78	1.7	200	37	4	44.9	81.1
		1.87	1.77	300	57	4.1	45.92	79.3
		1.98	1.79	400	75	4.44	49.28	73.9

Accordingly, we calculated the energy efficiency as follows:^70^
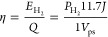
Table 2 shows the energy conversion efficiency at different temperatures and current densities. At a system temperature of 70 °C and a current density of 100 mA/cm^2^, the energy efficiency reaches 95.1%. The power loss does not include the thermal energy, as we hope to replace it with industrial waste heat or biomass-generated heat to improve the efficiency of hydrogen production through water electrolysis. Table 2 also shows the specific energy consumption per cubic meter or per kilogram of hydrogen; it decreases from 4.02 to 3.83 kWh/m^3^. These results demonstrate that NiMoO_4_/CP/APPJ-60s exhibits better OER performance in AEMWE, simultaneously improving the efficiency of hydrogen production through water electrolysis and achieving the effect of low-energy hydrogen production.

## Conclusion

NiMoO_4_ was grown on CP by a hydrothermal method. APPJ was then used for rapid surface processing to generate more oxygen-deficient NiMoO_4_ on the CP through plasma and ion bombardment. This enhanced the OER performance at the electrode. At 100 mA/cm^2^, both the overpotential and the Tafel slope decreased. Furthermore, the charge transfer resistance decreased while the EDLC increased, indicating lower electrode surface impedance and a larger ECSA. In AEMWE testing, the sample treated with APPJ for 60 s exhibited a higher current density at the same voltage. At a system temperature of 70 °C and a current density of 100 mA/cm^2^, the energy efficiency reached 95.1%. The specific energy consumption per kilogram of hydrogen produced decreased from 4.02 to 3.83 kWh/m^3^ after 60 s of APPJ treatment on NiMoO_4_/CP. These results demonstrate that APPJ-processed NiMoO_4_/CP offers excellent OER performance, thereby improving the water electrolysis efficiency in AEMWE systems. Consequently, it shows great potential to replace precious metal electrodes in large-scale water electrolysis systems.

## Supporting Information Available

Comparison of different OER electrocatalyst, APPJ temperature, SEM-EDS mapping images, ratio of different oxidation state, HRXPS spectra, LSV curve after stability test, electrochemical characteristics of the cathode material (Ru on CP) used in AEMWE, and comparative data of NiMoO_4_/CP and NiMoO_4_/CP-APPJ-60 s used in AEMWE at different operation temperatures (PDF)

## References

[ref1] Wang Q.; Hisatomi T.; Jia Q.; Tokudome H.; Zhong M.; Wang C.; Pan Z.; Takata T.; Nakabayashi M.; Shibata N.; Li Y.; Sharp I.; Kudo A.; Yamada T.; Domen K. Scalable Water Splitting on Particulate Photocatalyst Sheets With a Solar-to-hydrogen Energy Conversion Efficiency Exceeding 1. Nat. Mater. 2016, 15 (6), 611–615. 10.1038/nmat4589. 26950596

[ref2] Zainal B. S.; Ker P. J.; Mohamed H.; Ong H. C.; Fattah I. M. R.; Rahman S. M. A.; Nghiem L. D.; Mahlia T. M. I. Recent Advancement and Assessment of Green Hydrogen Production Technologies. Renew. Sustain. Energy Rev. 2024, 189, 113941 10.1016/j.rser.2023.113941.

[ref3] Yu M.; Wang K.; Vredenburg H. Insights Into Low-Carbon Hydrogen Production Methods: Green, Blue and Aqua Hydrogen. Int. J. Hydrogen Energy 2021, 46 (41), 21261–21273. 10.1016/j.ijhydene.2021.04.016.

[ref4] Miller H. A.; Bouzek K.; Hnat J.; Loos S.; Bernäcker C. I.; Weißgärber T.; Röntzsch L.; Meier-Haack J. Green Hydrogen From Anion Exchange Membrane Water Electrolysis: A Review of Recent Developments in Critical Materials and Operating Conditions. Sustain. Energy Fuels 2020, 4 (5), 2114–2133. 10.1039/C9SE01240K.

[ref5] Panchenko V. A.; Daus Y. V.; Kovalev A. A.; Yudaev I. V.; Litti Y. V. Prospects for The Production of Green Hydrogen: Review of Countries With High Potential. Int. J. Hydrogen Energy 2023, 48 (12), 4551–4571. 10.1016/j.ijhydene.2022.10.084.

[ref6] Qin Y.; Wang Z.; Yu W.; Sun Y.; Wang D.; Lai J.; Guo S.; Wang L. High Valence M-Incorporated PdCu Nanoparticles (M = Ir, Rh, Ru) for Water Electrolysis in Alkaline Solution. Nano Lett. 2021, 21 (13), 5774–5781. 10.1021/acs.nanolett.1c01581. 34187162

[ref7] Zhang K.; Zou R. Advanced Transition Metal-Based OER Electrocatalysts: Current Status, Opportunities, and Challenges. Small 2021, 17 (37), e2100129 10.1002/smll.202100129. 34114334

[ref8] Yuan S.; Duan X.; Liu J.; Ye Y.; Lv F.; Liu T.; Wang Q.; Zhang X. Recent Progress on Transition Metal Oxides as Advanced Materials for Energy Conversion And Storage. Energy Storage Mater. 2021, 42, 317–369. 10.1016/j.ensm.2021.07.007.

[ref9] Sharan H.; Madhavan J.; Mariappan G.; Kalai Selvan R.; Mani A. Unlocking the Electrocatalytic Behavior of Cu(2)MnS(2) Nanoflake-Anchored rGO for the Oxygen Evolution Reaction in an Alkaline Medium. Langmuir 2024, 40, 22230 10.1021/acs.langmuir.4c02824. 39394039

[ref10] Yoon K.-Y.; Lee K.-B.; Jeong J.; Kwak M.-J.; Kim D.; Roh H. Y.; Lee J.-H.; Choi S. M.; Lee H.; Yang J. Improved Oxygen Evolution Reaction Kinetics with Titanium Incorporated Nickel Ferrite for Efficient Anion Exchange Membrane Electrolysis. ACS Catal. 2024, 14 (7), 4453–4462. 10.1021/acscatal.3c05761.

[ref11] Cheng M.; Fan H.; Song Y.; Cui Y.; Wang R. Interconnected Hierarchical Nico(2)O(4) Microspheres as High-Performance Electrode Materials for Supercapacitors. Dalton Trans. 2017, 46 (28), 9201–9209. 10.1039/C7DT01289F. 28678249

[ref12] Zheng J.; Peng X.; Xu Z.; Gong J.; Wang Z. Cationic Defect Engineering in Spinel NiCo2O4 for Enhanced Electrocatalytic Oxygen Evolution. ACS Catal. 2022, 12 (16), 10245–10254. 10.1021/acscatal.2c01825.

[ref13] Jiang H.; Cui Z.; Xu C.; Li W. Humid Atmospheric Pressure Plasma Jets Exposed Micro-Defects On Comoo(4) Nanosheets with Enhanced OER Performance. Chem. Commun. (Camb) 2019, 55 (64), 9432–9435. 10.1039/C9CC04493K. 31328209

[ref14] Zhu J.; Qian J.; Peng X.; Xia B.; Gao D. Etching-Induced Surface Reconstruction of NiMoO_4_ for Oxygen Evolution Reaction. Nanomicro Lett. 2023, 15 (1), 30 10.1007/s40820-022-01011-3. 36624193 PMC9829944

[ref15] Bhat M. A.; Majid K. Metal-Organic Framework-Derived FeCo_2_S_4_/Co_3_O_4_ Heterostructure with Enhanced Electrocatalytic Performance for Oxygen Evolution and Hydrogen Evolution Reactions. Langmuir 2023, 39 (23), 8224–8233. 10.1021/acs.langmuir.3c00695. 37270702

[ref16] Liao H.; Zhang X.; Niu S.; Tan P.; Chen K.; Liu Y.; Wang G.; Liu M.; Pan J. Dynamic Dissolution and Re-Adsorption of Molybdate Ion in Iron Incorporated Nickel-Molybdenum Oxyhydroxide for Promoting Oxygen Evolution Reaction. Appl. Catal., B 2022, 307, 121150 10.1016/j.apcatb.2022.121150.

[ref17] Yang S.; Tiwari S. K.; Zhu Z.; Cao D.; He H.; Chen Y.; Thummavichai K.; Wang N.; Jiang M.; Zhu Y. In Situ Fabrication of Mn-Doped NiMoO(4) Rod-like Arrays as High Performance OER Electrocatalyst. Nanomater. 2023, 13 (5), 827 10.3390/nano13050827. PMC10005328 36903705

[ref18] Kim M.; Kim J.; Qin L.; Mathew S.; Han Y.; Li O. L. Gas-Liquid Interfacial Plasma Engineering Under Dilute Nitric Acid to Improve Hydrophilicity and OER Performance of Nickel Foam. Prog. Nat. Sci.: Mater. Int. 2022, 32 (5), 608–616. 10.1016/j.pnsc.2022.10.002.

[ref19] Rauscher T.; Bernäcker C. I.; Loos S.; Vogt M.; Kieback B.; Röntzsch L. Spark-Plasma-Sintered Porous Electrodes for Efficient Oxygen Evolution in Alkaline Water Electrolysis. Electrochim. Acta 2019, 317, 128–138. 10.1016/j.electacta.2019.05.102.

[ref20] Alers G. B.; Fleming R. M.; Wong Y. H.; Dennis B.; Pinczuk A.; Redinbo G.; Urdahl R.; Ong E.; Hasan Z. Nitrogen Plasma Annealing for Low Temperature Ta2O5 Films. Appl. Phys. Lett. 1998, 72 (11), 1308–1310. 10.1063/1.120569.

[ref21] Chou C.-Y.; Chang H.; Liu H.-W.; Yang Y.-J.; Hsu C.-C.; Cheng I. C.; Chen J.-Z. Atmospheric-Pressure-Plasma-Jet Processed Nanoporous Tio2photoanodes and Pt Counter-Electrodes for Dye-Sensitized Solar Cells. RSC Adv. 2015, 5 (57), 45662–45667. 10.1039/C5RA05014F.

[ref22] Liu H.-W.; Liang S.-p.; Wu T.-J.; Chang H.; Kao P.-K.; Hsu C.-C.; Chen J.-Z.; Chou P.-T.; Cheng I.-C. Rapid Atmospheric Pressure Plasma Jet Processed Reduced Graphene Oxide Counter Electrodes for Dye-Sensitized Solar Cells. ACS Appl. Mater. Interfaces 2014, 6 (17), 15105–15112. 10.1021/am503217f. 25127290

[ref23] Wang C.; Tian Y.; Gu Y.; Xue K.-H.; Sun H.; Miao X.; Dai L. Plasma-Induced Moieties Impart Super-Efficient Activity to Hydrogen Evolution Electrocatalysts. Nano Energy 2021, 85, 106030 10.1016/j.nanoen.2021.106030.

[ref24] Yu S.-E.; Wang Y.-C.; Tseng C.-Y.; Cheng I. C.; Chen J.-Z. Characteristics Of Low-Pressure-Plasma-Processed Niru-Mofs/Nickel Foam for Hydrogen Evolution Reaction. Phys. Scr. 2024, 99 (4), 045605 10.1088/1402-4896/ad314a.

[ref25] Su Y.-L.; Yu S.-E.; Ni I. C.; Wu C.-I.; Chen Y.-S.; Chuang Y.-C.; Cheng I. C.; Chen J.-Z. Low-Pressure Plasma-Processed NiCo Metal–Organic Framework for Oxygen Evolution Reaction and Its Application in Alkaline Water Electrolysis Module. J. Compos. Sci. 2024, 8 (1), 19 10.3390/jcs8010019.

[ref26] Chen J.-Z.; Wang C.; Hsu C.-C.; Cheng I. C. Ultrafast Synthesis of Carbon-Nanotube Counter Electrodes for Dye-Sensitized Solar Cells Using An Atmospheric-Pressure Plasma Jet. Carbon 2016, 98, 34–40. 10.1016/j.carbon.2015.10.078.

[ref27] Yu S.-E.; Su Y.-L.; Ni I.-C.; Chuang Y.-C.; Hsu C.-C.; Wu C.-I.; Chen Y.-S.; Cheng I.-C.; Chen J.-Z. Direct Current Pulse Atmospheric Pressure Plasma Jet Treatment on Electrochemically Deposited NiFe/Carbon Paper and Its Potential Application in an Anion-Exchange Membrane Water Electrolyzer. Langmuir 2024, 40 (29), 14978–14989. 10.1021/acs.langmuir.4c01169. 38946167 PMC11271009

[ref28] Qiu C.; Xu Z.; Chen F.-Y.; Wang H. Anode Engineering for Proton Exchange Membrane Water Electrolyzers. ACS Catal. 2024, 14 (2), 921–954. 10.1021/acscatal.3c05162.

[ref29] Furutani Y.; Shimizu Y.; Harada J.; Muto Y.; Yonezawa A.; Iguchi S.; Shida N.; Atobe M. Electrocatalytic Oxidation of Primary Alcohols at the Triple-Phase Boundary in an Anion-Exchange Membrane Reactor with Nickel, Cobalt, and Iron Catalysts. ACS Catal. 2024, 14 (11), 8922–8929. 10.1021/acscatal.4c01097.

[ref30] Chi J.; Yu H. Water Electrolysis Based on Renewable Energy for Hydrogen Production. Chin. J. Catal. 2018, 39 (3), 390–394. 10.1016/S1872-2067(17)62949-8.

[ref31] Feng Q.; Yuan X. Z.; Liu G.; Wei B.; Zhang Z.; Li H.; Wang H. A Review of Proton Exchange Membrane Water Electrolysis on Degradation Mechanisms and Mitigation Strategies. J. Power Sources 2017, 366, 33–55. 10.1016/j.jpowsour.2017.09.006.

[ref32] Guo W.; Kim J.; Kim H.; Han G. H.; Jang H. W.; Kim S. Y.; Ahn S. H. Sandwich-Like Co (OH) X/Ag/Co (OH) 2 Nanosheet Composites for Oxygen Evolution Reaction in Anion Exchange Membrane Water Electrolyzer. J. Alloys Compd. 2021, 889, 161674 10.1016/j.jallcom.2021.161674.

[ref33] Jeon S. S.; Lim J.; Kang P. W.; Lee J. W.; Kang G.; Lee H. Design principles Of Nife-Layered Double Hydroxide Anode Catalysts for Anion Exchange Membrane Water Electrolyzers. ACS Appl. Mater. Interfaces 2021, 13 (31), 37179–37186. 10.1021/acsami.1c09606. 34251792

[ref34] Han S.; Kim S.; Kim T. H.; Lee J. Y.; Yoon J. Optimizing the Synergistic Effect of Co and Fe for Efficient and Durable Oxygen Evolution under Alkaline Conditions. ACS Appl. Mater. Interfaces 2024, 16 (27), 35200–35207. 10.1021/acsami.4c07058. 38934926

[ref35] Kim S.; Min K.; Kim H.; Yoo R.; Shim S. E.; Lim D.; Baeck S.-H. Bimetallic-Metal Organic Framework-Derived Ni3S2/Mos2 Hollow Spheres as Bifunctional Electrocatalyst for Highly Efficient and Stable Overall Water Splitting. Int. J. Hydrogen Energy. 2022, 47 (13), 8165–8176. 10.1016/j.ijhydene.2021.12.208.

[ref36] Chen P.; Hu X. High-Efficiency Anion Exchange Membrane Water Electrolysis Employing Non-Noble Metal Catalysts. Adv. Energy Mater. 2020, 10 (39), 2002285 10.1002/aenm.202002285.

[ref37] Zhang B.; Shang X.; Jiang Z.; Song C.; Maiyalagan T.; Jiang Z.-J. Atmospheric-Pressure Plasma Jet-Induced Ultrafast Construction of an Ultrathin Nonstoichiometric Nickel Oxide Layer with Mixed Ni^3+^/Ni^2+^ Ions and Rich Oxygen Defects as an Efficient Electrocatalyst for Oxygen Evolution Reaction. Adv. Energy Mater. 2021, 4 (5), 5059–5069. 10.1021/acsaem.1c00623.

[ref38] Wang Y. C.; Yu S. E.; Su Y. L.; Cheng I. C.; Chuang Y. C.; Chen Y. S.; Chen J. Z. NiFe(2)O(4) Material on Carbon Paper as an Electrocatalyst for Alkaline Water Electrolysis Module. Micromachines (Basel) 2024, 15 (1), 62 10.3390/mi15010062. PMC10819468 38258181

[ref39] Senthil Raja D.; Cheng C. C.; Ting Y. C.; Lu S. Y. NiMo-MOF-Derived Carbon-Armored Ni(4)Mo Alloy of an Interwoven Nanosheet Structure as an Outstanding pH-Universal Catalyst for Hydrogen Evolution Reaction at High Current Densities. ACS Appl. Mater. Interfaces 2023, 15 (16), 20130–20140. 10.1021/acsami.3c01061. 36946987

[ref40] Tseng C.-H.; Hsin J.-C.; Tsai J.-H.; Chen J.-Z. Dielectric-Barrier-Discharge Jet Treated Flexible Supercapacitors with Carbon Cloth Current Collectors of Long-Lasting Hydrophilicity. J. Electrochem. Soc. 2020, 167 (11), 116511 10.1149/1945-7111/aba4e5.

[ref41] Gotoh K.; Yasukawa A. Atmospheric Pressure Plasma Modification of Polyester Fabric for Improvement of Textile-Specific Properties. Text. Res. J. 2011, 81 (4), 368–378. 10.1177/0040517510387207.

[ref42] Li Z.; Sun M.; Li Y.; Liu Z.; Zhang D.; Liu Y.; He X.; Sun M. J. NiMOF-Derived MoSe_2_/NiSe Hollow Nanoflower Structures as Electrocatalysts for Hydrogen Evolution Reaction in Alkaline Medium. Langmuir 2024, 40 (41), 21514–21523. 10.1021/acs.langmuir.4c02398. 39352217

[ref43] Tao L.; Duan X.; Wang C.; Duan X.; Wang S. Plasma-Engineered Mos2 Thin-Film as An Efficient Electrocatalyst for Hydrogen Evolution Reaction. Chem. Commun. (Camb) 2015, 51 (35), 7470–7473. 10.1039/C5CC01981H. 25829057

[ref44] Durr R. N.; Maltoni P.; Tian H.; Jousselme B.; Hammarstrom L.; Edvinsson T. From Nimoo(4) to Gamma-Niooh: Detecting The Active Catalyst Phase By Time Resolved In Situ And Operando Raman Spectroscopy. ACS Nano 2021, 15 (8), 13504–13515. 10.1021/acsnano.1c04126. 34383485 PMC8388116

[ref45] Thiagarajan K.; Bavani T.; Arunachalam P.; Lee S. J.; Theerthagiri J.; Madhavan J.; Pollet B. G.; Choi M. Y. Nanofiber NiMoO(4)/g-C(3)N(4) Composite Electrode Materials for Redox Supercapacitor Applications. Nanomater. 2020, 10 (2), 392 10.3390/nano10020392. PMC7075326 32102243

[ref46] Yang M.; Yang H.; Wang F.; Niu Y.; Li P. Synergistic Effects Boosting Hydrogen Evolution Performance of Transition Metal Oxides At Ultralow Ru Loading Levels. RSC Adv. 2023, 13 (19), 13263–13268. 10.1039/D3RA01501G. 37124022 PMC10141579

[ref47] Zhuang S.; Tong S.; Wang H.; Xiong H.; Gong Y.; Tang Y.; Liu J.; Chen Y.; Wan P. The P/Nife Doped Nimoo4Micro-Pillars Arrays for Highly Active And Durable Hydrogen/Oxygen Evolution Reaction Towards Overall Water Splitting. Int. J. Hydrogen Energy. 2019, 44 (45), 24546–24558. 10.1016/j.ijhydene.2019.07.138.

[ref48] Li Y.; Gao Y.; Yang S.; Wu C.; Tan Y. Anion-Modulated Nickel-Based Nanoheterostructures as High Performance Electrocatalysts for Hydrogen Evolution Reaction. J.Mater. Chem. 2020, 8 (24), 12013–12027. 10.1039/D0TA03513K.

[ref49] Yu C.; Liu Z.; Han X.; Huang H.; Zhao C.; Yang J.; Qiu J. Nico-Layered Double Hydroxides Vertically Assembled on Carbon Fiber Papers as Binder-Free High-Active Electrocatalysts for Water Oxidation. Carbon 2016, 110, 1–7. 10.1016/j.carbon.2016.08.020.

[ref50] Zhao X.; Meng J.; Yan Z.; Cheng F.; Chen J. Nanostructured Nimoo4 As Active Electrocatalyst For Oxygen Evolution. Chin. Chem. Lett. 2019, 30 (2), 319–323. 10.1016/j.cclet.2018.03.035.

[ref51] Zhang L.; Wu L.; Li J.; Lei J. Electrodeposition of Amorphous Molybdenum Sulfide Thin Film For Electrochemical Hydrogen Evolution Reaction. BMC Chem. 2019, 13 (1), 88 10.1186/s13065-019-0600-0. 31384835 PMC6661953

[ref52] Liu T.; Chai H.; Jia D.; Su Y.; Wang T.; Zhou W. Rapid Microwave-Assisted Synthesis of Mesoporous Nimoo4 Nanorod/Reduced Graphene Oxide Composites for High-Performance Supercapacitors. Electrochim. Acta 2015, 180, 998–1006. 10.1016/j.electacta.2015.07.175.

[ref53] Guo D.; Luo Y.; Yu X.; Li Q.; Wang T. High Performance Nimoo4 Nanowires Supported on Carbon Cloth as Advanced Electrodes for Symmetric Supercapacitors. Nano Energy 2014, 8, 174–182. 10.1016/j.nanoen.2014.06.002.

[ref54] Jain S.; Shah J.; Negi N. S.; Sharma C.; Kotnala R. K. Significance of Interface Barrier at Electrode of Hematite Hydroelectric Cell for Generating Ecopower by Water Splitting. Int. J. Energy Res. 2019, 43 (9), 4743–4755. 10.1002/er.4613.

[ref55] Karmakar A.; Karthick K.; Sankar S. S.; Kumaravel S.; Ragunath M.; Kundu S. Oxygen Vacancy Enriched Nimoo 4 Nanorods via Microwave Heating: A Promising Highly Stable Electrocatalyst for Total Water Splitting. J.Mater. Chem. 2021, 9 (19), 11691–11704. 10.1039/D1TA02165F.

[ref56] Ghosh D.; Pradhan D. Effect of Cooperative Redox Property and Oxygen Vacancies on Bifunctional OER and HER Activities of Solvothermally Synthesized CeO2/CuO Composites. Langmuir 2023, 39 (9), 3358–3370. 10.1021/acs.langmuir.2c03242. 36847346

[ref57] Wang D.; Wang J.; Luo X.; Wu Z.; Ye L. In Situ Preparation of Mo2C Nanoparticles Embedded in Ketjenblack Carbon as Highly Efficient Electrocatalysts for Hydrogen Evolution. ACS Sustain. Chem. Eng. 2018, 6 (1), 983–990. 10.1021/acssuschemeng.7b03317.

[ref58] Gopalakrishnan M.; Mohamad A. A.; Nguyen M. T.; Yonezawa T.; Qin J.; Thamyongkit P.; Somwangthanaroj A.; Kheawhom S. Recent Advances in Oxygen Electrocatalysts Based on Tunable Structural Polymers. Mater. Today Chem. 2022, 23, 100632 10.1016/j.mtchem.2021.100632.

[ref59] Sivanantham A.; Shanmugam S. Nickel Selenide Supported on Nickel Foam as An Efficient and Durable Non-Precious Electrocatalyst for The Alkaline Water Electrolysis. Appl. Catal., B 2017, 203, 485–493. 10.1016/j.apcatb.2016.10.050.

[ref60] Masa J.; Sinev I.; Mistry H.; Ventosa E.; de la Mata M.; Arbiol J.; Muhler M.; Roldan Cuenya B.; Schuhmann W. Ultrathin High Surface Area Nickel Boride (NixB) Nanosheets as Highly Efficient Electrocatalyst for Oxygen Evolution. Adv. Energy Mater. 2017, 7 (17), 1700381 10.1002/aenm.201700381.

[ref61] Suen N. T.; Hung S. F.; Quan Q.; Zhang N.; Xu Y. J.; Chen H. M. Electrocatalysis for The Oxygen Evolution Reaction: Recent Development and Future Perspectives. Chem. Soc. Rev. 2017, 46 (2), 337–365. 10.1039/C6CS00328A. 28083578

[ref62] Zhang K.; Zou R. Advanced Transition Metal-Based OER Electrocatalysts: Current Status, Opportunities, and Challenges. Small 2021, 17 (37), 2100129 10.1002/smll.202100129. 34114334

[ref63] Chen H.; Qiao S.; Yang J.; Du X. Nimo/Nico2o4 as Synergy Catalyst Supported on Nickel Foam for Efficient Overall Water Splitting. J. Mol. Catal. 2022, 518, 112086 10.1016/j.mcat.2021.112086.

[ref64] Rajput A.; Adak M. K.; Chakraborty B. Intrinsic Lability of NiMoO(4) to Excel the Oxygen Evolution Reaction. Inorg. Chem. 2022, 61 (29), 11189–11206. 10.1021/acs.inorgchem.2c01167. 35830301

[ref65] Alobaid A.; Wang C.; Adomaitis R. A. Mechanism and Kinetics of HER and OER on NiFe LDH Films in an Alkaline Electrolyte. J. Electrochem. Soc. 2018, 165 (15), J3395–J3404. 10.1149/2.0481815jes.

[ref66] Yin X.; Sun G.; Song A.; Wang L.; Wang Y.; Dong H.; Shao G. A Novel Structure of Ni-(Mos 2 /GO) Composite Coatings Deposited on Ni Foam under Supergravity Field As Efficient Hydrogen Evolution Reaction Catalysts In Alkaline Solution. Electrochim. Acta 2017, 249, 52–63. 10.1016/j.electacta.2017.08.010.

[ref67] Zheng X.; Yang Z.; Wu J.; Jin C.; Tian J.-H.; Yang R. Phosphorus and Cobalt Co-Doped Reduced Graphene Oxide Bifunctional Electrocatalyst for Oxygen Reduction and Evolution Reactions. RSC Adv. 2016, 6 (69), 64155–64164. 10.1039/C6RA12438K.

[ref68] Wang H.; Wang Z.; Feng Z.; Qiu J.; Lei X.; Wang B.; Guo R. Application Progress of Nimoo4 Electrocatalyst in Basic Oxygen Evolution Reaction. Catal. Sci. Technol. 2024, 14 (3), 533–554. 10.1039/D3CY01514A.

[ref69] Xiao Z.; Wang J.; Liu C.; Wang B.; Zhang Q.; Xu Z.; Sarwar M. T.; Tang A.; Yang H. In-Situ Surface Structural Reconstruction of NiMoO_4_ for Efficient Overall Water Splitting. Appl. Surf. Sci. 2022, 602, 154314 10.1016/j.apsusc.2022.154314.

[ref70] Dawood F.; Anda M.; Shafiullah G. M. Hydrogen Production for Energy: An Overview. Int. J. Hydrogen Energy. 2020, 45 (7), 3847–3869. 10.1016/j.ijhydene.2019.12.059.

