# Supplementary material for: Enhanced Oxygen Evolution Reaction Performance of NiMoO4/Carbon Paper Electrocatalysts in Anion Exchange Membrane Water Electrolysis by Atmospheric-Pressure Plasma Jet Treatment
Source: Langmuir. 2024 Nov 1;40(46):24675–86. doi: 10.1021/acs.langmuir.4c03557 (PMC11580383; doi:10.1021/acs.langmuir.4c03557)
Supplement: Supplementary file 1 — la4c03557_si_001.pdf [file la4c03557_si_001.pdf]

Supplementary Information

Enhanced Oxygen Evolution Reaction Performance of $NiMoO_4$/Carbon Paper Electrocatalysts in Anion Exchange Membrane Water Electrolysis by Atmospheric-Pressure Plasma Jet Treatment

Chen-Chen Chueh[1], Shuo-En Yu[1], Hsing-Chen Wu[2] , Cheng-Che Hsu[3], I-Chih Ni[4], Chih-I Wu[1,4], I-Chun Cheng[4], Jian-Zhang Chen[1,2,5,]*

[1] Graduate School of Advanced Technology, National Taiwan University, Taipei City 106319, Taiwan

[2] Institute of Applied Mechanics, National Taiwan University, Taipei City 106319, Taiwan

[3] Department of Chemical Engineering, National Taiwan University, Taipei City 106319, Taiwan

[4]Department of Electrical Engineering and Graduate Institute of Photonics and Optoelectronics, National Taiwan University, Taipei City 106319, Taiwan

[5] Advanced Research Center for Green Materials Science and Technology, National Taiwan University, Taipei City 106319, Taiwan

**Correspondence: jchen@ntu.edu.tw*

List

Table S1. Comparison of the OER electrocatalyst performance in alkaline solution in literature.

Figure S1. APPJ temperature versus time

Figure S2. Experimental setup for AEMWE system.

Figure S3. (a) $NiMoO_4$/CP-APPJ-60 s_3000X and (b)–(e) SEM-EDS elemental mapping images of $NiMoO_4$/CP-APPJ-60 s.

Table S2. Area ratio of different oxidation states in Ni 2p.

Table S3. Area ratio of different oxidation states in Mo 3d.

Table S4. Area ratio of different oxidation states in O 1s.

Figure S4. HRXPS spectra of C 1s: **(a)** $NiMoO_4$/CP, **(b)** $NiMoO_4$/CP/APPJ-30s, **(c)** $NiMoO_4$/CP/APPJ-60s, and **(d)** $NiMoO_4$/CP/APPJ-90s.

Table S5. Area ratio of different oxidation states in C 1S.

Figure S5. LSV polarization curves of $NiMoO_4$/CP/APPJ-60s after 24 h stability test.

Figure S6. LSV polarization curves of $NiMoO_4$/CP/APPJ-60s after 24 h stability test.

Figure S7. Power supply voltage and current density curves in the AEMWE at different temperatures: **(a)** $NiMoO_4$/CP(+)|Ru/CP/LPP-60s(-) and **(b)** $NiMoO_4$/CP/APPJ-60s (+)|Ru/CP/LPP-60s(-).

Figure S8. Comparison of different anode electrocatalysts in AEMWE: **(a)** room temperature and **(b)** 70 °C.

Table S1. Comparison of the OER electrocatalyst performance in alkaline solution in literature.

Electrocatalyst	Electrolyte	Overpotential (mV)	Reference
**$NiMoO_4$/CP/APPJ-60 s**	**1 M KOH**	**368@10**<br>**790@100**	**This work**
**$CoMoO_4$-60 s**	1 M KOH	314@10	[1]
**P-CC**	1 M KOH	450@10	[2]
**$NiFe_2O4$(QDs)/CNTs**	1 M KOH	450@10	[3]
**N-HPCS@Co1 Cu1 Fe NSs**	1 M KOH	460@10	[4]
**Mo_4S_{16}@GCA**	1 M KOH	370@10	[5]
**Co0.89Ca0.11-CPs**	1 M KOH	371@10	[6]
**Mn-Fe oxide/CP**	0.1 M KOH	670@10	[7]

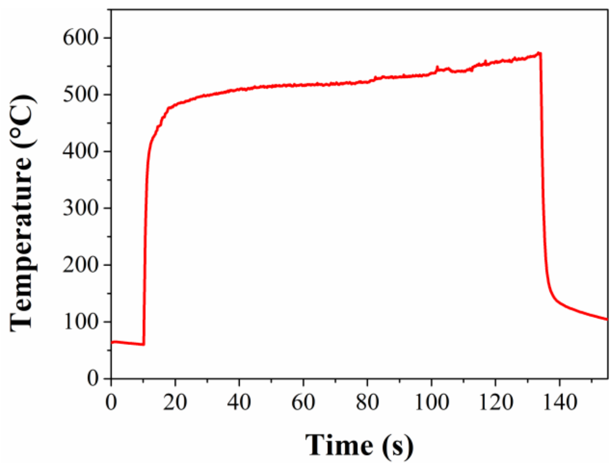

Figure S1. APPJ temperature versus time

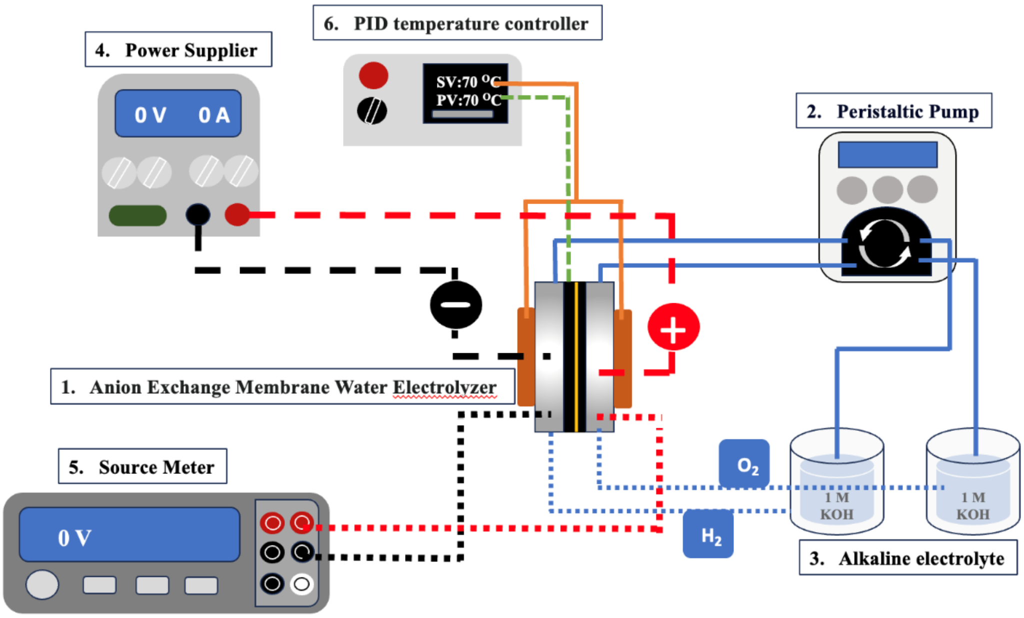

Figure S2. Experimental setup for AEMWE system.

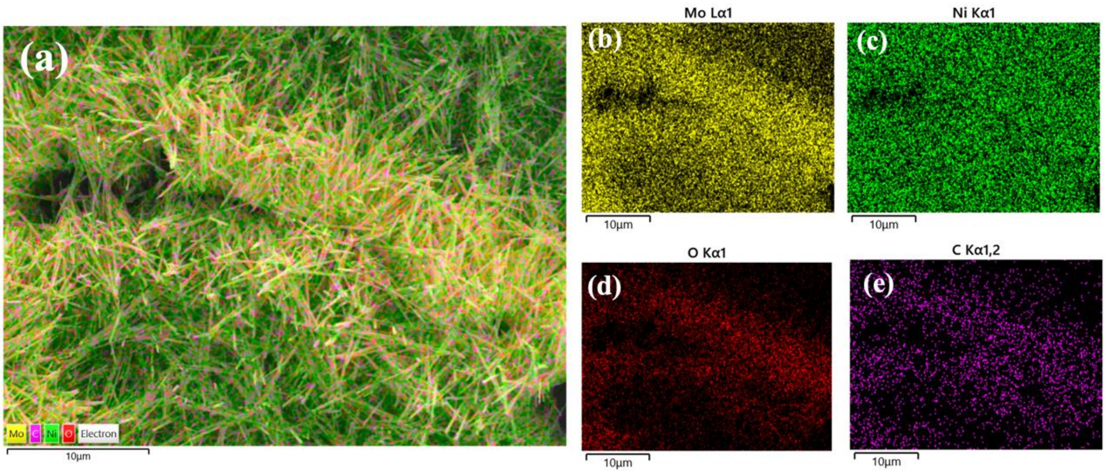

Figure S3. (a) $NiMoO_4$/CP-APPJ-60 s_3000X and (b)–(e) SEM-EDS elemental mapping images of $NiMoO_4$/CP-APPJ-60 s.

Table S2. Area ratio of different oxidation states in Ni 2p.

**Area (%)**	**Ni^{2+}**	**Ni^{3+}**	**Sat.**	**Ni^{2+}/Ni^{3+}**
**$NiMoO_4$/CP**	2.55	24.92	72.53	0.102
**$NiMoO_4$/CP/APPJ-30 s**	11.64	33.35	55.01	0.35
**$NiMoO_4$/CP/APPJ-60 s**	**30.04**	**12.53**	**57.43**	**2.4**
**$NiMoO_4$/CP/APPJ-90 s**	30.1	11.97	57.93	2.51

Table S3. Area ratio of different oxidation states in Mo 3d.

**Area (%)**	**Mo^{4+}**	**Mo^{5+}**	**Mo^{6+}**	**Mo^{6+}/Mo**
**$NiMoO_4$/CP**	10.44	83.33	6.23	6.23
**$NiMoO_4$/CP/APPJ-30 s**	0	93.5	6.5	6.5
**$NiMoO_4$/CP/APPJ-60 s**	**0**	**86.35**	**13.65**	**13.65**
**$NiMoO_4$/CP/APPJ-90 s**	0	92.07	7.93	7.93

Table S4. Area ratio of different oxidation states in O 1s.

**Area (%)**	**M-O-M**	**Oxygen vacancy**	**H_2O**
**$NiMoO_4$/CP**	**40.6**	**28.5**	**30.9**
**$NiMoO_4$/CP/APPJ-60s**	**30.3**	**49.7**	**20**

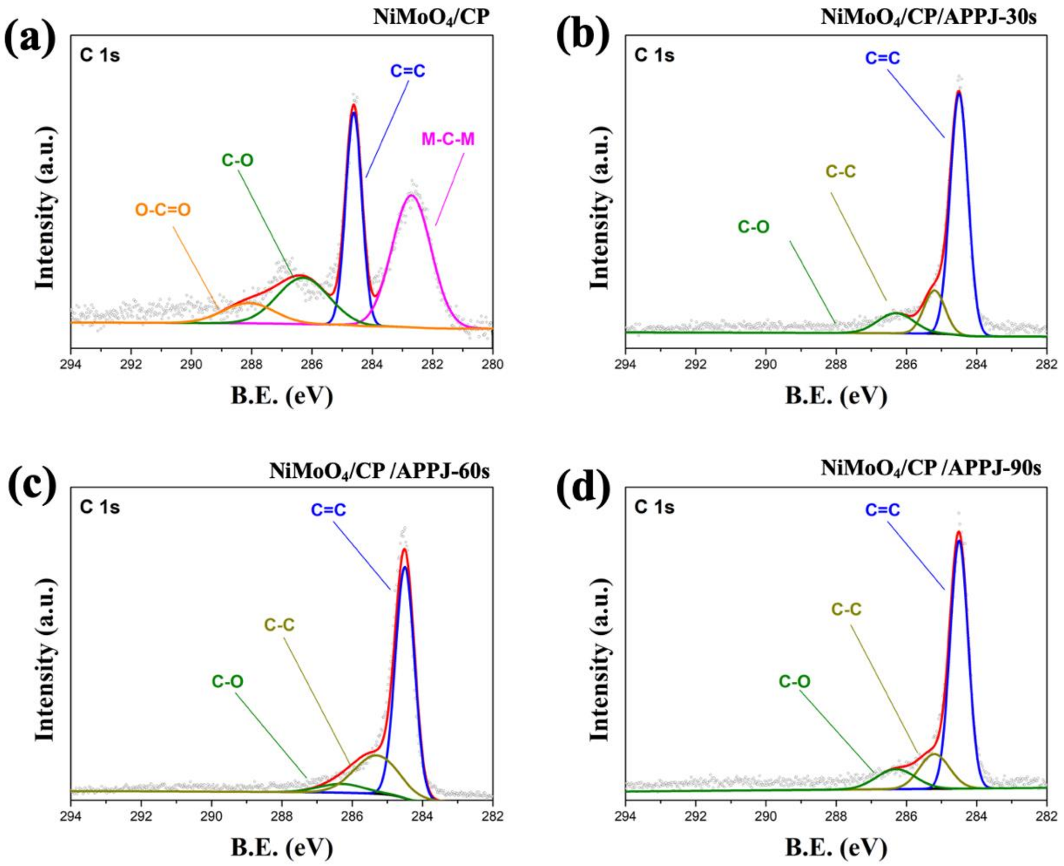

Figure S4. HRXPS spectra of C 1s: **(a)** $NiMoO_4$/CP, **(b)** $NiMoO_4$/CP/APPJ-30s, **(c)** $NiMoO_4$/CP/APPJ-60s, and **(d)** $NiMoO_4$/CP/APPJ-90s.

Table S5. Area ratio of different oxidation states in C 1S.

Area (%)	C=C	M-C-M	C-C	C-O	O-C=O
**$NiMoO_4$/CP**	28.64	42.8	0	19.81	8.7
**$NiMoO_4$/CP/APPJ-30 s**	72.7	0	15.2	12.1	0
**$NiMoO_4$/CP/APPJ-60 s**	67.5	0	26.4	6.05	0
**$NiMoO_4$/CP/APPJ-90 s**	71	0	16.6	12.4	0

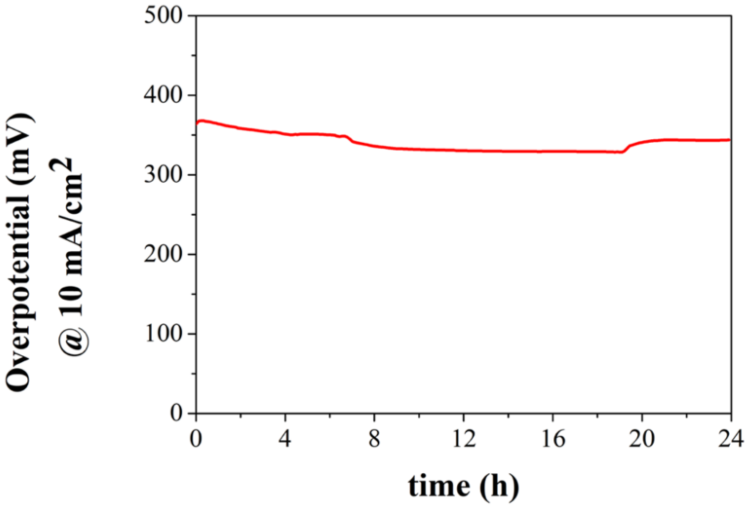

Figure S5. Stability test of $NiMoO_4$/CP/APPJ-60 s.

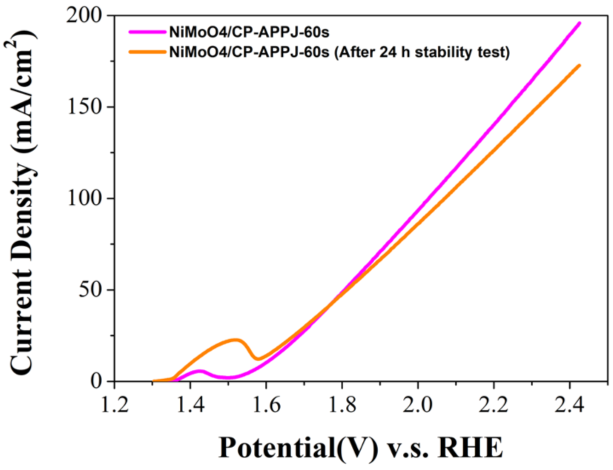

Figure S6. LSV polarization curves of $NiMoO_4$/CP/APPJ-60s after 24 h stability test.

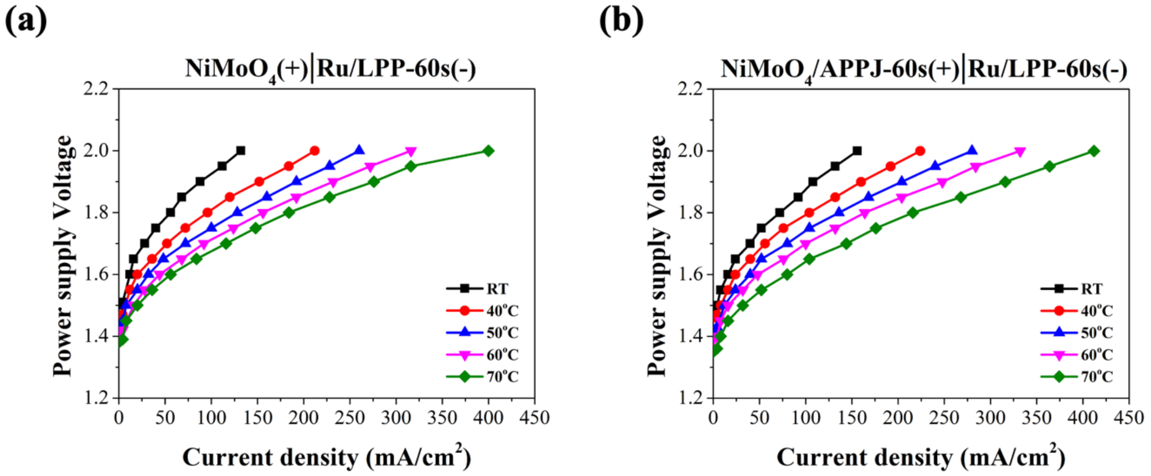

Figure S7. Power supply voltage and current density curves in the AEMWE at different temperatures: **(a)** $NiMoO_4$/CP(+)|Ru/CP/LPP-60s(-) and **(b)** $NiMoO_4$/CP/APPJ-60s (+)|Ru/CP/LPP-60s(-).

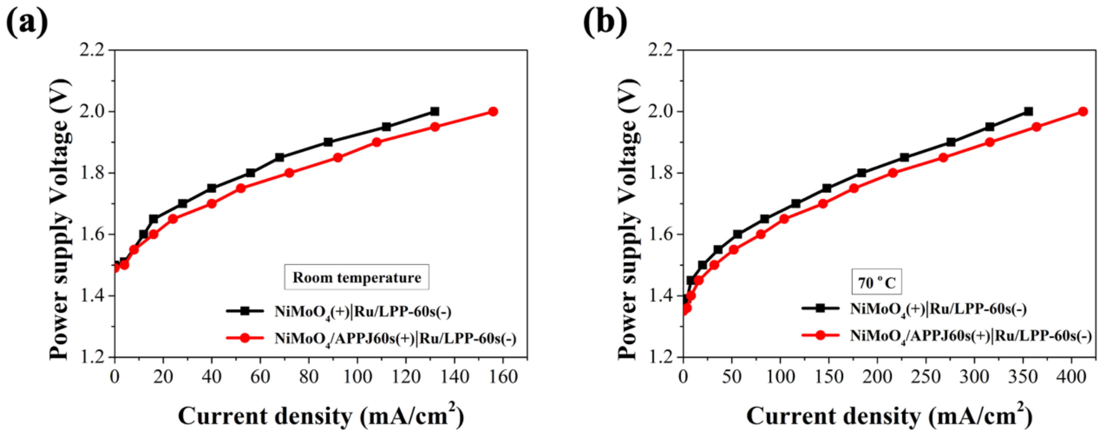

Figure S8. Comparison of different anode electrocatalysts in AEMWE: **(a)** room temperature and **(b)** 70 °C.

Reference

1. Jiang, H.; Cui, Z.; Xu, C.; Li, W. *Humid Atmospheric Pressure Plasma Jets Exposed Micro-Defects on Comoo(4) Nanosheets With Enhanced OER Performance.* Chem. Commun. (Camb.), 2019. **55**(64): p. 9432-9435.
2. Liu, Z.; Zhao, Z.; Wang, Y.; Dou, S.; Yan, D.; Liu, D.; Xia, Z.; Wang, S. *In Situ Exfoliated, Edge-Rich, Oxygen-Functionalized Graphene from Carbon Fibers for Oxygen Electrocatalysis.* Adv Mater, 2017. **29**(18).
3. Xu, N.; Zhang, Y.; Zhang, T.; Liu, Y.; Qiao, J. *Efficient Quantum Dots Anchored Nanocomposite for Highly Active ORR/OER Electrocatalyst of Advanced Metal-Air Batteries.* Nano Energy, 2019. **57**: p. 176-185.
4. Liu, J.; Ning, G.; Shi, K.; Zheng, M.; Sun, Y.; Gao, Y.; Zhang, Y.; Wang, H. *N-Doped Hollow Porous Carbon Spheres@Co Cu Fe Alloy Nanospheres as Novel Non-Precious Metal Electrocatalysts for HER And OER.* Int. J. Hydrogen Energy., 2022. **47**(9): p. 5947-5960.
5. Cheng, Y.; Yuan, P.; Xu, X.; Guo, S.; Pang, K.; Guo, H.; Zhang, Z.; Wu, X.; Zheng, L.; Song, R. *S-Edge-Rich Mo(X)S(Y) Arrays Vertically Grown on Carbon Aerogels as Superior Bifunctional HER/OER Electrocatalysts.* Nanoscale, 2019. **11**(42): p. 20284-20294.
6. Su, P.; Ma, S.; Hung, W.; Boyjoo, Y.; Bai, S.; Liu, J. *Ca2+-Doped Ultrathin Cobalt Hydroxyl Oxides Derived from Coordination Polymers as Efficient Electrocatalysts for The Oxidation of Water.* J.Mater. Chem., 2019. **7**(33): p. 19415-19422.
7. Bhandary, N.; Ingole, P. P.; Basu, S. *Electrosynthesis of Mn-Fe Oxide Nanopetals on Carbon Paper As Bi-Functional Electrocatalyst for Oxygen Reduction and Oxygen Evolution Reaction.* Int. J. Hydrogen Energy, 2018. **43**(6): p. 3165-3171.
